# Ultrafast and Radiation-Hard Lead Halide Perovskite Nanocomposite Scintillators

**DOI:** 10.1021/acsenergylett.3c01396

**Published:** 2023-08-28

**Authors:** Andrea Erroi, Sara Mecca, Matteo L. Zaffalon, Isabel Frank, Francesco Carulli, Alessia Cemmi, Ilaria Di Sarcina, Doriana Debellis, Francesca Rossi, Francesca Cova, Kristof Pauwels, Michele Mauri, Jacopo Perego, Valerio Pinchetti, Angiolina Comotti, Francesco Meinardi, Anna Vedda, Etiennette Auffray, Luca Beverina, Sergio Brovelli

**Affiliations:** † Dipartimento di Scienza dei Materiali, Università degli Studi Milano - Bicocca, via R. Cozzi 55, 20126 Milan, Italy; ‡ CERN, Esplanade des Particules 1, 1211 Meyrin, Switzerland; § LMU Munich, Geschwister-Scholl-Platz 1, 80539 Munich, Germany; ∥ ENEA Fusion and Technology for Nuclear Safety and Security Department, Casaccia R.C., Via Anguillarese 301, 00123 Rome, Italy; ⊥ Electron Microscopy Facility, Istituto Italiano di Tecnologia, 16163 Genova, Italy; # IMEM-CNR Institute, Parco Area delle Scienze 37/A, 43124 Parma, Italy; ∇ ESRF - The European Synchrotron, 71 Avenue des Martyrs, 38000 Grenoble, France

## Abstract

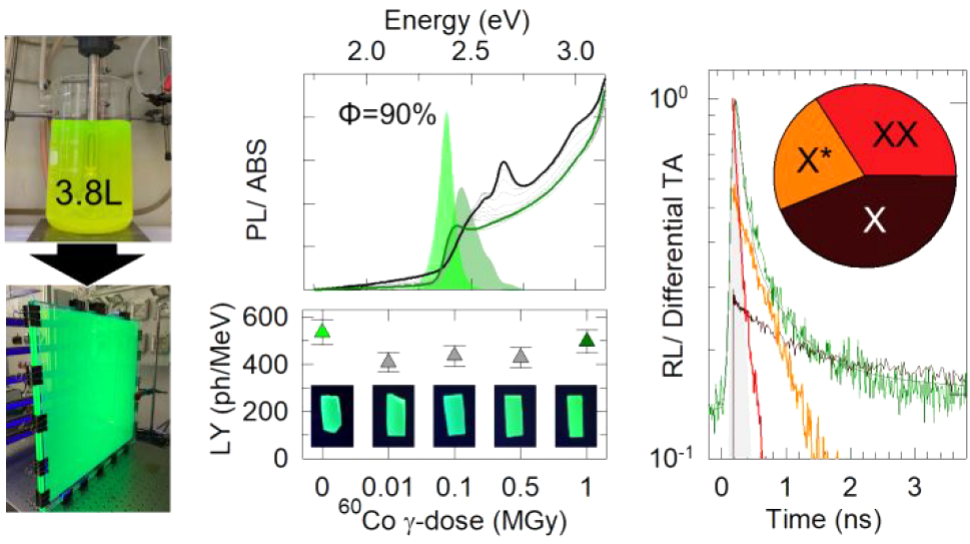

The use of scintillators for the detection of ionizing radiation is a critical aspect in many fields, including medicine, nuclear monitoring, and homeland security. Recently, lead halide perovskite nanocrystals (LHP-NCs) have emerged as promising scintillator materials. However, the difficulty of affordably upscaling synthesis to the multigram level and embedding NCs in optical-grade nanocomposites without compromising their optical properties still limits their widespread use. In addition, fundamental aspects of the scintillation mechanisms are not fully understood, leaving the scientific community without suitable fabrication protocols and rational guidelines for the full exploitation of their potential. In this work, we realize large polyacrylate nanocomposite scintillators based on CsPbBr_3_ NCs, which are synthesized via a novel room temperature, low waste turbo-emulsification approach, followed by their in situ transformation during the mass polymerization process. The interaction between NCs and polymer chains strengthens the scintillator structure, homogenizes the particle size distribution and passivates NC defects, resulting in nanocomposite prototypes with luminescence efficiency >90%, exceptional radiation hardness, 4800 ph/MeV scintillation yield even at low NC loading, and ultrafast response time, with over 30% of scintillation occurring in the first 80 ps, promising for fast-time applications in precision medicine and high-energy physics. Ultrafast radioluminescence and optical spectroscopy experiments using pulsed synchrotron light further disambiguate the origin of the scintillation kinetics as the result of charged-exciton and multiexciton recombination formed under ionizing excitation. This highlights the role of nonradiative Auger decay, whose potential impact on fast timing applications we anticipate via a kinetic model.

The detection of high-energy photons (X or γ), particles (α, β) and neutrons, commonly referred to as ionizing radiation, is at the heart of many strategic applications in both science and technology,^1^ including high-energy/particle physics,^2^ space exploration,^3^ medical diagnostics,^4−6^ cargo screening,^7^ border security,^8^ and industrial and environmental monitoring.^9,10^ Typically, ionizing radiation is detected using direct radiation-to-charge converters^11,12^ or scintillator materials^13−15^ which emit UV–visible photons upon interaction with ionizing radiation by physical processes dependent on the nature of the radiation itself, such as Coulomb collisions, Compton scattering, photoelectric effect, and carrier pair formation.^16^ The fundamental characteristics of a scintillator are the probability of interaction with ionizing radiation, which scales with the *n*th power of the average atomic number *Z* (where *n* = 1–5 depending on the type of interaction),^17,18^ the scintillation efficiency or light yield (LY), expressed as the number of photons emitted per unit of absorbed energy, and the stability at high doses of absorbed radiation, also known as radiation hardness.^19−22^ The scintillation rate is of paramount importance when radiation detection is performed in time-of-flight (TOF) mode, which assigns a precise time tag to each scintillation event.^23^ In particle physics, TOF techniques are essential for discriminating rare events in high-luminosity accelerators, where picosecond fast detection is required to mitigate the effects of signal build-up and to identify event peaks.^24^ Precise time tagging is also used in time-of-flight positron emission tomography (TOF-PET) to improve image spatial resolution and signal-to-noise ratio to accurately distinguish small neoplastic formations in oncology, as well as in neurological, rheumatological, infectious, and cardiological diagnosis.^25−30^ Specifically, TOF-PET scanners use the coincident detection of two 511 keV photons emitted 180° apart in the same direction to reconstruct the map of electron-positron annihilation events caused by a radiopharmaceutical tracer in the body. The time delay between the arrival of the two γ-photons is proportional to the difference in their path lengths and thus contains information about the spatial location where the annihilation process took place. It follows that any improvement in the so-called coincidence time resolution (CTR)^31^ has a direct beneficial effect on the resolution of TOF-PET images, thus motivating the technology race toward a CTR < 10 ps, corresponding to millimeter resolution, which represents a more than 10-fold improvement compared to state-of-the-art commercial TOF-PET scanners.^23^ Finally, for rapid industrial technology transfer and large-scale applications, it is essential that scintillators can be manufactured in large sizes and/or quantities using methods that are affordable in terms of both process energy and raw materials.^32^

Recently, so-called nanocomposite scintillators based on high-*Z* scintillator nanocrystals (NC) embedded in polymeric matrices have emerged as promising alternatives to traditional materials,^33^ such as inorganic scintillator crystals—which are prohibitively expensive and energy-intensive and cannot be produced in large sizes/volumes^19^—or plastic scintillators,^34^ which can be produced cheaply in large sizes and customized shapes but are radiation-soft^22^ and have lower energy resolution.^35^ By exploiting the efficient and fast scintillation of NCs^36,37^ in combination with the flexibility of plastic fabrication, nanocomposite scintillators hold promise to bridge the gap between the single crystal and plastic approaches, thus enabling a leap forward in radiation detection schemes. In particular, lead halide perovskites (LHP) NCs, in the inorganic and hybrid forms APbX_3_ (where A is methylammonium, formamidinium, or Cs and X is a halogen), have recently emerged as promising nanoscintillators^11,14,38−40^ valued for their tunable fast and efficient scintillation^41^ and unique tolerance to structural defects,^42,43^ enabling competitive LY and radiation hardness up to extreme radiation levels,^44^ comparable to the annual dose accumulated in nuclear reactors or high-brightness particle accelerators.^45,46^ Despite these advantages, the widespread use of nanocomposite scintillators based on LHP-NCs has been hampered mainly by manufacturing constraints. In particular, hot-injection synthesis methods^47,48^ used for high-optical-quality LHP NCs are not suitable for mass production, and more scalable ligand assisted reprecipitation (commonly referred to as LARP)^49−51^ techniques, in which NCs growth is initiated by the addition of antisolvents at room temperature, may suffer from concentration gradients in the reaction environment (especially at multi-liter scale) resulting in generally poorer optical performance.^52,53^ The recently reported kinetically controlled synthesis of spheroidal LHP NCs by Akkerman et al. offers exciting possibilities for further development in this respect.^54^ However, a common problem with wet syntheses of LHP-NCs is the use of excess reagents and the formation of byproducts that generally result in the amount of waste produced exceeding the product by orders of magnitude. In the case of LHP NCs, the problem is particularly severe due to the non-negligible contamination with toxic lead compounds. Although considerable efforts are being made to develop protocols for the recovery of lead from spent LHP-based devices, only a few studies^55,156^ have been reported for the recycling of the waste generated during NCs synthesis. Finally, the compatibility of LHP NCs with optical polymeric matrices, such as polyacrylates (e.g., poly(methyl methacrylate)) or polystyrenes (e.g., polystyrene, poly vinyl toluene) is typically limited by their ionic nature: acrylate monomers can be mass polymerized using room temperature photoradical processes, but their polarity damages the NC surfaces.^56,57^ Monomers that do not affect the NCs such as styrene, on the other hand, require thermal polymerization approaches at high *T* (≥80 °C)^58−60^ that degrade the NCs and/or cause crystallinity transitions to nonemissive phases.^61,62^ Direct synthesis of NCs by thermal annealing of polymer blends containing LHP precursors has also been explored,^63^ but the slow nucleation of supersaturated precursors in the residual solvents often leads to uncontrolled NC size and aggregation, resulting in poor optical quality and cloudy samples even at very low NC loadings.^64^ As a result, most LHP-NCs composite scintillators reported to date have been prepared by solvent evaporation from NC/polymer solutions, which produces useful model systems but is incompatible with large scale manufacturing.^33,41^ To date, the fabrication of efficient LHP-NCs nanocomposite scintillators by scalable and affordable means remains an open challenge.

Here, we aim to contribute to this effort by realizing large, ultrafast, optical-grade nanocomposite scintillators based on high-emissivity CsPbBr_3_ NCs synthesized by a high-throughput, multigram-scale turbo-emulsified method followed by their in situ passivation during the polymerization process. This fast and low-waste approach offers significant advantages over conventional methods. Namely, the turbo-emulsifier homogenized synthesis of CsPbBr_3_ NCs, performed here for the first time, is inherently large scale and cost-effective, and in contrast to common behavior, the polymer embedding almost completely suppresses nonradiative quenching channels and unifies the NC population in the final product. Low boiling and highly volatile solvents are used in the synthesis, making their separation by distillation and the further recovery of unreacted species effective and energy efficient. As a result, large scale nanocomposites (60 × 50 × 0.3 cm^3^) with photoluminescence (PL) quantum yields up to Φ_PL_ = 90%, spectrally pure narrow excitonic radioluminescence (RL) with LY up to ∼4800 photons/MeV even at relatively low NC loadings (0.8 wt %), and radiation hardness up to extreme irradiation conditions (up to 1 MGy ^60^Co γ-ray dose) can be fabricated inexpensively at room temperature, and the recovered residue can be used to synthesize a new batch of NCs with comparable optical properties. Fundamentally for radiation detection, time-resolved RL experiments show that the scintillation of our nanocomposites is ultrafast, with over 30% of the emitted photons radiated faster than 80 ps, resulting in a potential CTR in the range of about 39 to 90 ps with 511 keV gamma *excitation*,^65^ a very promising figure for TOF-PET applications. Transient absorption (TA) measurements performed in parallel with time-resolved PL excited by pulsed synchrotron radiation up to 40 eV (more than six times the ionization potential of CsPbBr_3_)^66,67^ allow us to unambiguously assign the scintillation kinetics to multiexciton and charged-exciton decay dominated by nonradiative Auger recombination (AR). Finally, numerical simulations of decay kinetics in the biexciton regime suggest that CTR is essentially independent of AR, which quenches and accelerates scintillation to a comparable extent. In addition to their technological relevance, these results elucidate important fundamental aspects of scintillation in nanostructured composites and suggest a viable strategy for the mass fabrication of ultrafast, radiation-resistant nanocomposites for advanced, fast-time radiation detection schemes.

To realize large size nanocomposites embedding CsPbBr_3_ NCs in a single multigram process, PbBr_2_ and tetrabutylammonium bromide (TBAB) (1:1 mol ratio) were dissolved at 80 °C in a mixture of oleylamine (540 mmol), propionic acid (540 mmol), and isopropanol (60 mL). After complete dissolution, the mixture was cooled down to room temperature. A solution of Cs_2_CO_3_ (6 mmol) in propionic acid (6 mL) was also prepared and diluted in 3.6 L of a heptane/isopropanol solution (2:1 in volume). The latter was put under turbo-emulsifier homogenization (15k rpm) and the first solution was swiftly added, inducing a rapid change from colorless to bright yellow. The mixture was allowed to homogenize for 30 s. The final crude solution looked clear and brightly green luminescent under ambient illumination, highlighting the formation of CsPbBr_3_ NCs (Figure 1a, hereafter referred to as *native* NCs). The crude solution was washed with 1.8 L of isopropanol and centrifuged at 4500 rpm for 2 min. The final weight of the dry product was ∼8 g. The process allows for the recycling of both the volatile solvents and the nonvolatile residue, as detailed in a separate section of the Supporting Information. Scanning transmission electron micrographs (STEM) of the product revealed the formation of a mixture of particles with different aspect ratios, as commonly observed for large batch room temperature synthesis processes (size, 13 ± 3 nm; thickness, 5 ± 1 nm; Figure 1b), with a predominance of rectangular particles as highlighted by the respective high-resolution TEM image in the inset of Figure 1b and the STEM overviews in Figure S1. The dried NCs were dispersed in a mixture of methyl methacrylate/lauryl methacrylate (MMA/LMA, 80:20 wt %) monomers with the addition of 2,2-dimethoxy-2-phenylacetophenone (0.33 wt %) that acts as photoinitiator (using 365 nm light) for the radical mass polymerization of an optical-grade random PMMA/PLMA copolymer^68^ unaffected by macroscopic phase segregation. The choice of PMMA as the main host material was dictated by its excellent optical properties and good radiation hardness,^22,44,69^ as further confirmed herein, that make it one of the main polymeric materials for fabricating optical components^70^ and in scintillator fibers.^22,71^ In turn, LMA with its long alkyl side chains creates a near-native nonpolar polymeric environment for the NCs, improving their miscibility and preserving their optical properties.^72^Figure 1c reports a photograph of a fabricated nanocomposite with dimensions of 60 × 50 × 0.3 cm comprising [NC] = 0.2 wt %. Fundamentally, the polymerization reaction not only led to the formation of a mechanically solid matrix but also played an active role for the evolution of the native NC mixture to a uniform ensemble of CsPbBr_3_ NCs with nearly perfect emission efficiency directly in a polymer host. TEM images of 70 nm thin cut nanocomposite slices (Figure 1d and Figure S2) and corresponding EDS elemental analysis performed in ADF-STEM mode (Figure S3) show nanoplatelet-like CsPbBr_3_ NCs (side, 14 ± 3 nm; thickness, 9 ± 2 nm) mostly concentrated in larger nanodomains, which suggests a particle ripening/merging process that occurs during the solidification of the polymer host, in agreement with recent results on CsPbBr_3_ nanowires-to-nanocubes transformation.^73^ The powder X-ray diffraction (PXRD) patterns of the native NCs and of the NCs embedded in the nanocomposite are reported in Figure 1e, and they correspond in both cases to the orthorhombic phase of CsPbBr_3_ and indicate that the NCs crystal structure is preserved after the polymerization reaction of the host matrix. The evolution of the native NCs during nanocomposite formation is rendered possibly more evident by *in situ* monitoring of their optical properties (Figure 1f–h).

**Figure 1:**
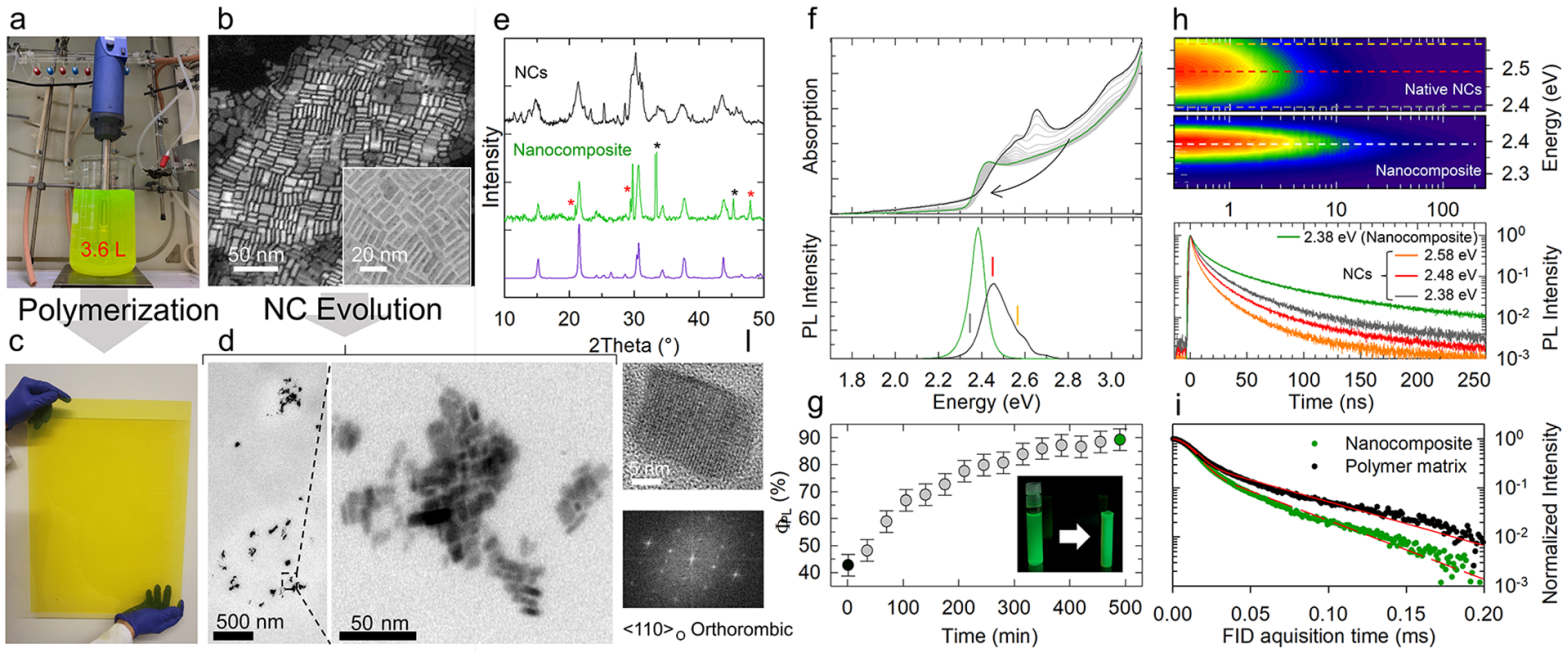
(a) Photograph of the turbo-emulsifier homogenized synthesis of 8 g of CsPbBr_3_ NCs in a 5 L reactor. (b) STEM-HAADF image of the NC sample after washing. Inset: corresponding HRTEM image. (c) Photograph of a fabricated PMMA–PLMA nanocomposite with dimensions of 60 × 50 × 0.3 cm comprising CsPbBr_3_ [NC] = 0.2 wt % under ambient illumination. (d) TEM micrographs of 70 nm thin nanocomposite section showing domains of CsPbBr_3_ NCs in the polymeric matrix. (e) Powder X-ray diffraction patterns of the native NCs (top, black line) and of the NCs embedded in the nanocomposite displayed in panel c (middle, green line), together with the calculated PXRD pattern for orthorhombic CsPbBr_3_ (bottom, purple line, ICSD 97851). The diffraction halo pattern associated with the polymeric host matrix in the PXRD of the nanocomposite was subtracted for clarity (Figure S6). The peaks denoted by star symbols are associated with minor crystalline impurities included in the nanocomposite (red stars, CsBr). (f) Optical absorption (top panel) and PL (bottom panel, excitation energy 3.1 eV) of 0.2 wt % dispersions of CsPbBr_3_ NCs in LMA:MMA (20:80%vol) during the polymerization reaction (time evolution indicated by the black arrow). The initial spectra before the activation of the UV initiators and at the end of the process are highlighted in black and green, respectively. (g) Evolution of the PL quantum yield was observed during the polymerization process. The photographs of the liquid monomer mixture and the polymerized solid under UV illumination (3.4 eV) are reported as inset. (h) Contour plot of the spectrally resolved PL decay traces of the native NCs (top panel) and the final nanocomposite (middle panel) excited at 3.1 eV and (bottom panel) representative decay curves collected at the emission energies indicated by the dashed lines in the contour plots and the vertical bars in panel f. (i) ^1^H NMR free induction decay (FID) at 333 K indicating faster relaxation for the nanocomposite (green dots, 0.2 wt %) with respect to the bare polymer matrix (black dots). The solid red lines are the fitting functions. (l) HRTEM image (top panel) and corresponding Fast Fourier Transform (FFT) pattern (bottom panel) of CsPbBr_3_ NCs evolved in a solution of acrylate monomers.

The optical absorption and PL spectra of the initial monomer dispersion (*t* = 0 min) featured multiple contributions at ∼2.4, 2.5, and 2.6 eV, and a substantial scattering tail consistent with the coexistence of different sized/shaped particles (Figure 1f). The convolution of multiple contributions in the emission profile was further confirmed by the spectral analysis of the PL decay traces in Figure 1h, showing faster decay kinetics for the blue side of the PL spectrum. Strikingly, during the polymerization of the acrylate matrix, the absorption spectrum progressively evolved toward the typical profile of CsPbBr_3_ NCs, with a prominent first excitonic peak at 2.4 eV, matching well the average particle thickness of 9 ± 2 nm^74,75^ extracted from the TEM analysis of the nanocomposite (Figure S2). The isosbestic point at 2.45 eV in Figure 1f confirmed the conversion of the heterogeneous population of native particles into a homogeneous NCs ensemble. Crucially for our purposes, NCs evolution led to substantial improvements of both the emission spectral purity and efficiency. As shown in Figure 1f–h, the PL efficiency monotonically increased from Φ_PL_ = 43 ± 5% to 90 ± 5%, and the final nanocomposites featured narrow PL (FWHM = 17 nm, 78 meV) with identical PL lifetime, *τ*_PL_ = 11 ns (as extracted from the time after which the PL intensity had dropped by a factor *e*) across the whole spectrum, around twice as long as any emission contribution by the native NCs. Based on the near unity Φ_PL_ and the very low excitation fluence (corresponding to average exciton occupancy ⟨*N*⟩ ∼ 0.01), we ascribe the observed PL lifetime to the radiative decay of single excitons. The whole body of spectroscopic data in Figure 1 indicates the gradual passivation of nonradiative losses likely associated with surface electron traps by the nonbonding electron pairs of the oxygen atoms of the polyacrylate chains.^76^ Time domain ^1^H NMR measurements acquired with the MSE refocusing block produced the fast-relaxing free induction decay (FID) expected for polymers below the glass transition. As shown in Figure 1i, the decay is faster in the nanocomposite, indicating a reduction in the local mobility of the polymer chains in the presence of the NCs. The Gaussian contribution associated with a fully rigid fraction extracted by bimodal fitting quantitatively demonstrate an increased rigid fraction from 59 ± 1% to 66 ± 1% in the nanocomposite, which is a typical consequence of the decrease of polymer chain motions due to attractive interactions with the NCs.^77^ Here, it is a further indication of the strong affinity between the NCs and the polymer matrix, which contributes to avoiding aggregation and obtaining a homogeneous dispersion. To further investigate the possible roles of the polymerization initiators or UV light in the NC evolution process, we monitored the structural and optical properties of native NCs dispersed in the monomer mixture without initiators or UV illumination over time. As shown in Figure 1 and in Figure S4 the NCs ensembles evolved very similarly, resulting in orthorhombic structured particles with the characteristic optical spectra of CsPbBr_3_ NCs and enhanced PL efficiency, thus suggesting that the gradual increase in the viscosity of the nanocomposite plays a negligible role in the process.

Based on the promising optical properties of our nanocomposites, we proceeded with investigating their scintillation and radiation hardness properties. In Figure 2a we show the RL spectra of five nanocomposites containing increasing loadings of CsPbBr_3_ NC ([NC] = 0.05 → 0.8 wt %; the respective transmission spectra are reported in Figure S5). In each case, the RL spectra were sharp single peaks due to the band-edge excitonic transition, thus further confirming the absence of structural defects that have been shown to cause spurious low-energy spectral contributions.^44^ The absolute LY of the five nanocomposites obtained using monochromatic 15 keV X-ray excitation and confirmed by side-by-side comparison with the commercial plastic scintillator EJ273D excited with a Bremsstrahlung distribution of X-rays with similar mean energy (∼7.2 keV) are reported in Figure 2b showing progressively higher LY with increasing [NC] reaching LY = 4800 ph/MeV for the 0.8 wt % composite.

**Figure 2:**
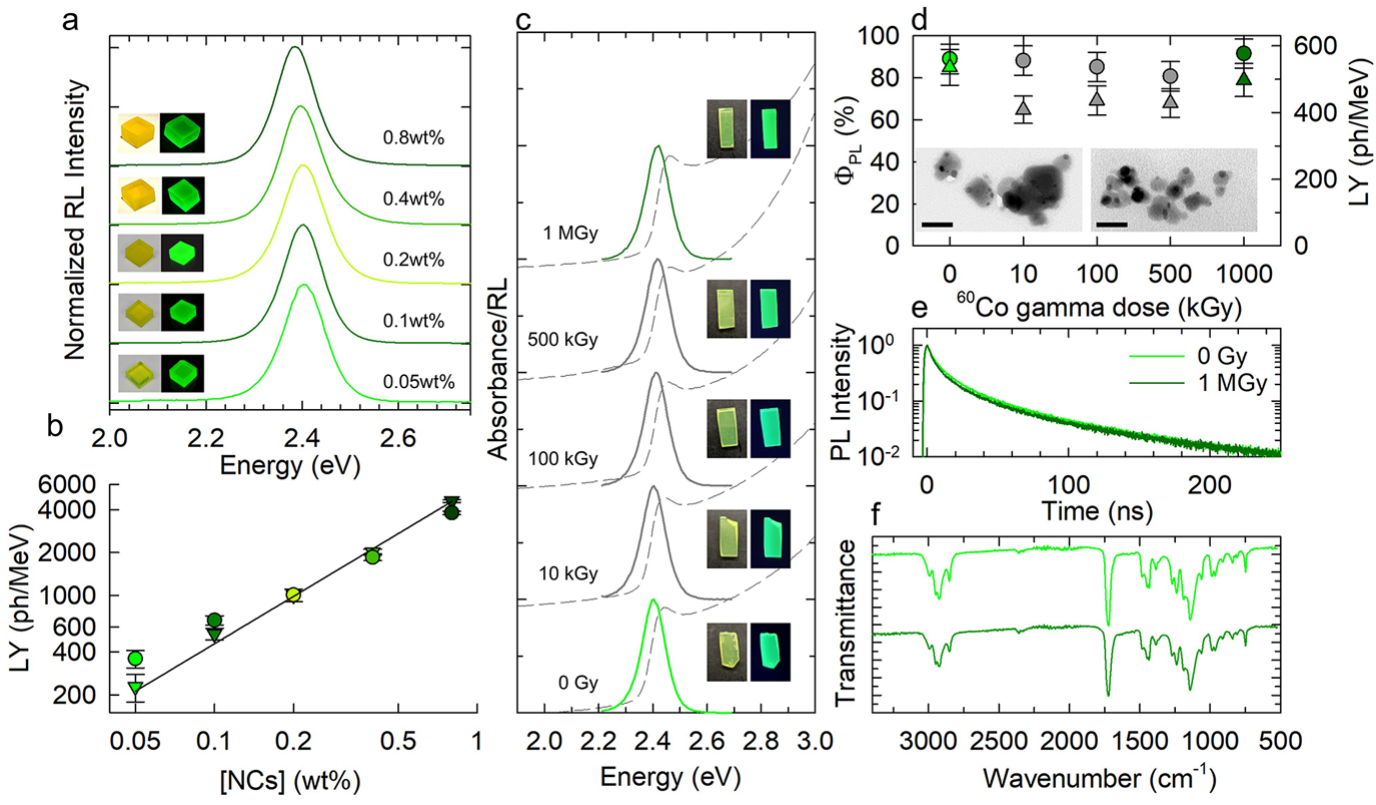
(a) RL spectra of polyacrylate nanocomposites containing increasing concentration of CsPbBr_3_ NCs together with respective photographs under ambient or UV light. (b) Corresponding LY values obtained via absolute (triangles) or relative (circles) methods. The black line is the fitting function with a power law *I*_RL_ = A × [NC]^*p*^ with *p* = 1.1. (c) Optical absorption (dashed lines), RL spectra (solid lines) and photographs under ambient or UV light of polyacrylate nanocomposites with [NC] = 0.1 wt % at increasing cumulative γ-ray doses from 0 Gy to 1 MGy (bottom to top). The spectra have been normalized at the emission maxima and respective absorption edge and vertically shifted for clarity. (d) LY (circles) and corresponding Φ_PL_ (triangles) as a function of cumulative dose. Inset: TEM micrographs of 70 nm thin nanocomposite sections before and after irradiation (scale bar 50 nm). (e) Normalized PL decay curves and (f) and FTIR transmission spectra at 0 Gy and 1 MGy showing no variation of the NC decay kinetics and no modification of the vibrational spectrum of the polymer indicating high radiation resistance.

Fundamentally, such LY and spectral properties were perfectly retained even after exposure to extremely high radiation levels comparable to the yearly γ-dose accumulated by the inner walls of a nuclear reactor or by the inner magnetic coil of the Large Hadron Collider.^45,46^ Specifically, to assess the radiation hardness of our systems, we used the Calliope irradiation facility (see the irradiation certification in the Supporting Information)^78^ to expose nanocomposite samples to uniform γ-ray irradiation by a ^60^Co source at a dose rate of 3.05 kGy_air_ h^–1^ and monitored their optical and scintillation properties at increasing cumulative doses up to as much as 1 MGy. The linear attenuation coefficient (μ) of CsPbBr_3_ at the mean γ-ray emission energy (1.25 MeV) of ^60^Co is μ = 0.261 cm^–1^, corresponding to a mass attenuation coefficient, μ/ρ = 5.376 × 10^–2^ cm^2^ g^–1^, where ρ is the density.^79^ For the tested nanocomposite containing 0.1 wt % of NCs, μ/ρ = 6.185 × 10^–2^ cm^2^ g^–1^ and μ = 7.008 × 10^–2^ cm^–1^. The fraction of energy deposited in the nanocomposite by the single γ photon is 2%, whereas the cumulative dose is 2.3 MGy, corresponding to 1.4 × 10^21^ eV. Remarkably, the optical absorption and RL spectra were perfectly preserved at all accumulated doses (Figure 2c) and so were the respective efficiencies and PL dynamics (Figure 2d,e and Figure S7 for NCs dispersed in the monomeric solution), thus demonstrating the stability of the nanocomposites even at extremely high radiation doses. The hardness is also corroborated by the TEM images reported as inset of Figure 2d and showing similar morphologies before and after 1 MGy irradiation. We emphasize that such resistance to ionizing radiation is not due to their relatively low density, as conventional plastic scintillators based on organic dyes with comparable attenuation coefficients and densities typically undergo strong quenching at much lower gamma doses.^22^ A possible cause of such a remarkable property could be found in the self-healing ability of lead halide perovskites after structural damage, such as the creation of vacancies or surface segregation of metallic lead, as recently reported by Milotti et al.^80^ As a further confirmation of the radiation hardness of the polymeric matrix, the Fourier transform infrared spectra of a pristine and a 1 MGy-irradiated nanocomposite shown in Figure 2f are essentially identical, with no peaks emerging after irradiation,^81^ suggesting negligible radiation induced damage even at huge radiation doses.

Next, we focused on the timing performance of our nanocomposites by performing scintillation kinetic measurements (scintillation rise and decay times) in time-correlated single photon counting (TCSPC) mode under X-ray excitation using the experimental configuration schematically depicted in Figure 3a. The scintillation decay curves of the five nanocomposites shown in Figure 2a are also reported in Figure 3a along with their fitting curves to a convolution of the IRF (FWHM = 160 ps) and the intrinsic scintillation rate. Notably, for all samples, we observed a prompt ultrafast decay component modeled with a Gaussian function (relative weight R_P_ ∼ 30% ) and a *τ*_1_ ∼ 0.6 ns long decay component with comparable weight followed by a longer-lived tail of around *τ*_2_ ∼ 10 ns matching the respective PL lifetime, *τ*_PL_. The time constant, relative weights and corresponding effective scintillation lifetime, τ_EFF_ extracted as the weighted harmonic average of the decay contributions, are summarized in Table 1 for the five nanocomposites and match well the timing performance recently reported for CsPbBr_3_ NCs synthesized by conventional hot-injection method and incorporated into a polystyrene host by solvent evaporation.^41^ The ultrafast timing capability demonstrated, in particular, by the prompt kinetic component is of great relevance for TOF technologies. Combining scintillation decay time and light output measurements, the potential time resolution reachable by these prototypes may be calculated from the following equation:^31^ (where *N* is the estimated number of emitted photons for a 511 keV excitation), resulting in an estimated time resolution in the range of about 39 to 90 ps with 511 keV gamma *excitation*, a very promising value for TOF-PET applications.

**Figure 3:**
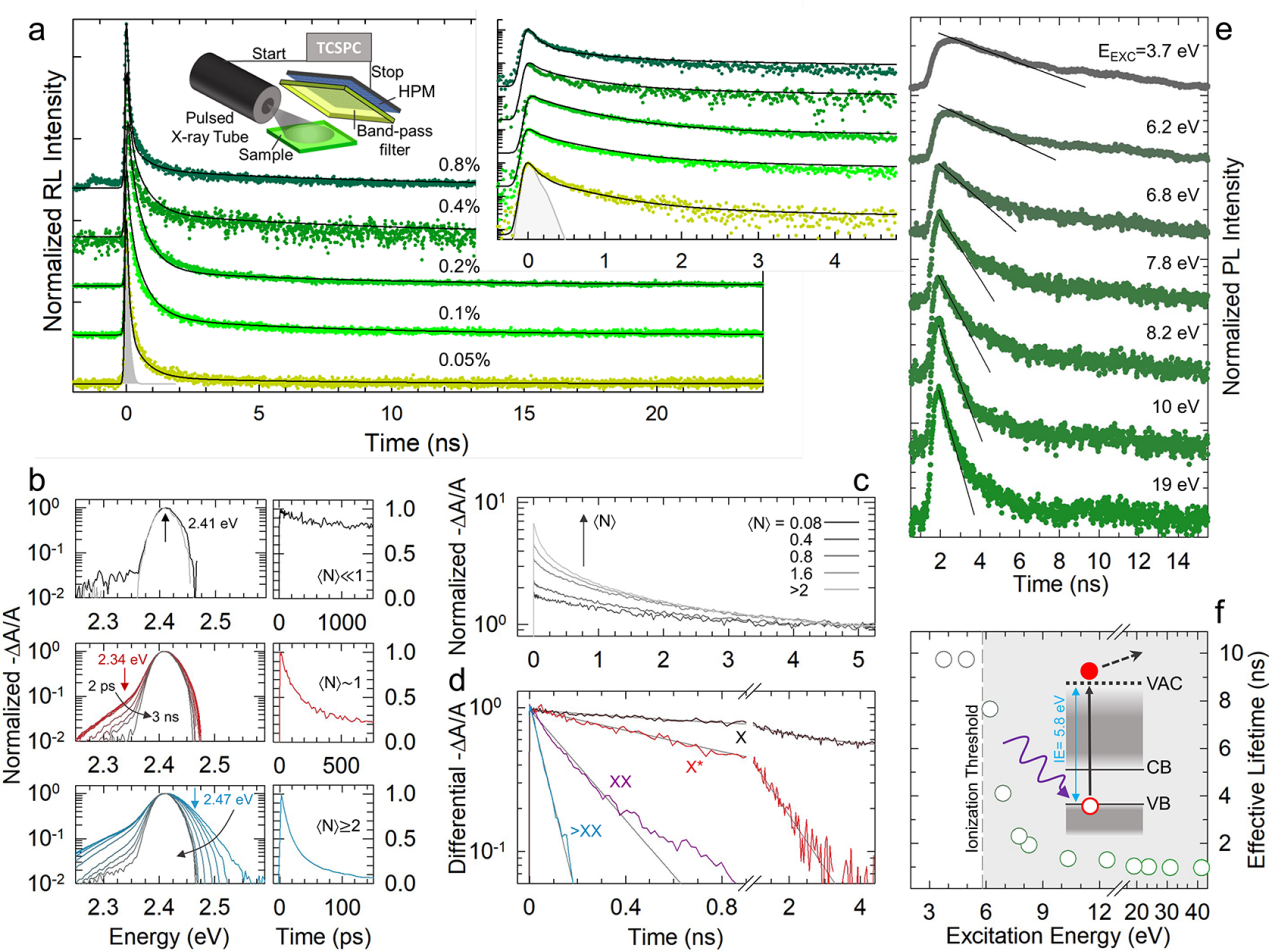
(a) Scintillation decay of the five nanocomposites shown in Figure 2a. The scintillation decay is shown in a linear scale over 24 ns. The inset shows details of the ultrafast component (over 5 ns) in semilogarithmic scale; the shaded gray line represents the system IRF. The solid curves are the fit functions. (b) Transient absorption spectra (in semilogarithmic scale) at increasing time (*t* = 2, 7, 12, 23, 50, 200, 500, 1000, 3000 ps) after the excitation pulse for progressively larger average exciton population ⟨*N*⟩ showing the emergence of bi- and multiexciton spectral contributions.^182^ The respective decay traces taken at the energies indicated in the figure are shown in the right-hand panels highlighting gradually faster decay with increasing ⟨*N*⟩. (c) TA dynamics at increasing average exciton population ⟨*N*⟩. (d) Differential TA curves extracted from panel c representing single-order, charged-order, bi-order, and higher order exciton dynamics. (e) PL decay traces excited by synchrotron light at increasing energy up to *E*_EXC_ = 19 eV together with the respective single exponential fitting curves. (f) Corresponding PL decay times vs *E*_EXC_. Inset: schematic depiction of the ionization of CsPbBr_3_ NCs upon excitation with the *E*_EXC_ ≥ IE.

**Table 1:** Decay Times (τ, ns) and Respective Relative Weights (*R*) Extracted from the Fitting of the RL Decays in Figure 3a^a^

[NC], wt %	*R*_P_	*R*_1_	τ_1_, ns	*R*_2_	τ_2_, ns	τ_EFF_, ns	LY, ph/MeV	*N* Estimated at 511 keV	CTR Estimated (ps)
0.05	0.30	0.37	0.61	0.33	22	1.13	238	121	93
0.1	0.32	0.21	0.62	0.47	8.7	1.76	489	250	81
0.2	0.34	0.22	0.60	0.44	6.8	1.54	1056	541	51
0.4	0.29	0.09	0.58	0.62	10.3	3.3	2014	1007	55
0.8	0.30	0.07	0.62	0.63	10.5	4.1	4800	2323	39

a For extracting the effective scintillation lifetime, the fit function was normalized so that the weights of the three components add up to one (*R*_P_ + *R*_1_ + *R*_2_ = 1), but the effective decay time was calculated with the re-normalized ratio of components τ_1_ and τ_2_ according to τ_EFF_ = (*R*_1n_/τ_1_ + *R*_2n_/τ_2_)^−1^, *R*_in_ = *R*_*i*_/(*R*_1_ + *R*_2_).^27^ For the estimation of the CTR, the risetime has been set to 84 ps, corresponding to the time needed for the IRF amplitude to increase from 10% to 90% of the total signal.

Besides the potential applicative performance of our CsPbBr_3_ NCs-based composites, it is relevant for future material optimization to identify the nature of the emissive states responsible for ultrafast RL dynamics. Previous studies on CdSe nanoplatelets by Turtos and co-workers^37^ ascribed ultrafast scintillation components (<200 ps) to the decay of multiexcitons generated by highly energetic X-ray excitation, in line with pulsed cathodoluminescence studies by the Schaller group on quantum dots of the same composition,^82^ yet for LHP NCs such an ascription is still debated. To disambiguate the origin of scintillation contributions between multiexcitonic and trapping processes^39^ in our CsPbBr_3_ NCs nanocomposites, we probed (multi)excitonic kinetics using transient absorption (TA) experiments as a function of increasing excitation fluence. The normalized TA spectra for the 0.2 wt % nanocomposite collected at increasing delay time after the excitation pulse (2 ps to 3 ns) at progressively higher excitation fluence—corresponding to the single exciton (X, average exciton occupancy ⟨*N*⟩ ≪ 1), biexciton (XX, ⟨*N*⟩ ≥ 1), and higher order multiexciton (MX, ⟨*N*⟩ > 2) regimes—are reported in Figure 3b. In the X regime, the TA spectrum showed the characteristic peak at 2.41 eV due to bleaching of the 1S exciton absorption at all times. The corresponding time dynamics was essentially single exponential with characteristic time, *τ*_X_ = 10 ns, which matches well the corresponding *τ*_PL_ = 11 ns, measured at vanishingly low excitation power, thus confirming that the bleach recovery is due to radiative decay of 1S excitons. In agreement with previous reports showing predominant attractive character of XX in CsPbBr_3_ NCs,^83,84^ for ⟨*N*⟩ ≥ 1 the TA spectra showed a low-energy shoulder at 2.34 eV, ∼80 meV below the main peak, with ∼200 ps decay time.^84^ Consistent with that, the TA dynamics developed an initial ultrafast decay portion characteristic of biexciton decay limited by Auger recombination (AR, Figure 3c),^85^ whose amplitude followed the characteristic ⟨*N*⟩^2^ trend of the Poisson biexciton state-filling statistics (Figure S8). Upon increasing the excitation fluence further, the TA spectra showed a contribution on the high-energy side of the 1S bleach peak with decay time of ∼80 ps, which we ascribe to higher order multiexcitons in line with previous observations on CsPbBr_1.5_I_1.5_ NCs^86^ under intense optical pumping. We further examined the (multi)excitonic dynamics following the procedure introduced by Klimov et al.,^85^ where the TA curves vs ⟨*N*⟩ were first normalized to their slow single exciton tail and then progressively subtracted to each other. The extracted TA decay curves are shown in Figure 3d together with the X trace measured for ⟨*N*⟩ ≪ 1 for direct comparison. For ⟨*N*⟩ > 1, the differential TA curves accelerated substantially with respect to the single exciton kinetics, yielding a biexciton lifetime *τ*_XX_ ∼ 200 ps. Considering a biexciton radiative lifetime,^85^*τ*_XX,Rad_ = τ_X,Rad_/4, and a single exciton radiative lifetime of *τ*_X,Rad_ = 10 ns, we obtain a that the picosecond XX decay is largely dominated by AR, with a biexciton yield Φ_XX_ = *τ*_XX_/*τ*_XX,Rad_ ∼ 0.08 consistent with similar CsPbBr_3_ materials.^86−93^ Further increasing the excitation fluence to ⟨*N*⟩ > 2 resulted in higher order multiexciton lifetime as short as *τ*_MX_ ∼ 60 ps, in agreement with recent reports on CsPbBr_3_ NCs of comparable size.^41^ For intermediate excitation fluence (⟨*N*⟩ ≤ 1), the bleach kinetics featured a lifetime of ∼0.8 ns, matching the intermediate timing component of the RL kinetics (*τ*_1_). To investigate the origin of such a component, we used pulsed synchrotron excitation at DESY laboratories to monitor the evolution of the PL dynamics of our nanocomposites (0.2 wt %) up to excitation energies largely above the ionization energy of CsPbBr_3_ (IE = 5.6–5.8 eV see refs (66), (94), and (95)). The PL decays excited up to 40 eV and the respective lifetimes extracted from single exponential fitting are shown in Figure 3e,f. For below-IE excitation, the PL kinetics showed an identical ∼10 ns contribution to the single exciton bleach dynamics (Figure 3d) and independently measured τ_PL_ using 3.1 eV optical excitation (Figure 1i). Notably, when *E*_EXC_ reached ∼5.8 eV, the PL lifetime underwent steep acceleration to τ ∼ 0.9 ns, matching the intermediate RL lifetime and TA dynamics for ⟨*N*⟩ ≤ 1 (notice that the IRF for the synchrotron excited time-resolved measurements was ∼800 ps). For higher *E*_EXC_ values, the PL lifetime remained constant. The close match between the onset of such an acceleration and the ionization energy of CsPbBr_3_ suggests that the ∼0.6 ns decay component is possibly due to photocharged NCs following the release of an electron from the top of the valence band occurring by photoelectric effect under ionizing excitation or via AR-mediated photoionization under intense optical pumping.

The similarity between the ultrafast RL components and the multiexciton TA dynamics is a clear indication that the scintillation timing of CsPbBr_3_ NCs is dominated by the decay of multiexcitons and not by nonradiative trapping processes (which are also effectively suppressed in our composites) as previously hypothesized. This has profound implications for the general understanding of scintillation in LHP NCs and suggests a role of AR in both the kinetics and efficiency of the scintillation process, which ultimately combine in the applicative timing capability expressed by the CTR. To clarify this aspect, it is instructive to compare the behavior of an ideal two-level system, such as NCs in the X regime, with that of NCs containing multiple excitons. In the first case, the effect of nonradiative channels (with lifetime *τ*_NR_) on the CTR,^27^ approximately described as 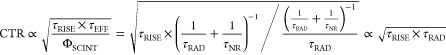
is relatively straightforward, as the acceleration effect by nonradiative processes on the effective emission lifetime, τ_EFF_ ∝ , is linearly compensated by the concomitant decrease of the scintillation quantum yield, Φ_SCINT_ ∝ , resulting in constant CTR values determined by the product of the rise and radiative lifetimes. The effect of AR on the scintillation kinetics and Φ_SCINT_ is, on the other hand, not as trivial because *i*) “slow emitting” X and “fast emitting” XX species inherently coexist in the multiexciton regime (based on Poissonian filling of quantized states) and *ii*) radiative and nonradiative (e.g., AR) decay of XX generate X, resulting in the dynamic conversion of fast emitting species into slow emitting ones.^85,86,96^ The schematic depiction of the fate of X and XX species is sketched in Figure 4a in the approximation that AR is the sole nonradiative process active in the system—i.e., neglecting multiphonon decay or trapping, which corresponds to Φ_PL_ = 1. Although the rigorous treatment of the scintillation process would require accounting for the exact energy deposition and (multi)exciton formation processes in a complex inhomogeneous composite material,^97^ helpful insights of the effect of AR on the timing performance can be gathered by simulating the decay kinetics described by the rate equations:
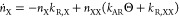
1
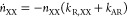
2where *n*_X_ (*n*_XX_) and *k*_R__,X_ (*k*_R__,XX_) are the X (XX) populations and radiative rates and *k*_AR_ is the biexciton AR rate. The positive term in eq 1 describes the formation of X from the decay of XX, either radiatively or via AR with efficiency Θ, where (1 – Θ) is the probability of particle ionization by carrier ejection or by trapping of hot carriers, as it occurs in so-called B-type blinking.^98^ In Figure 4b,c we show the simulated emission kinetics for Θ = 1 (nonionizing AR) and Θ = 0 (ionizing AR) obtained by solving eq 1 and eq 2 (see Supporting Information and Figure S9) as a function of the AR quantum yield, . For this simulation, we opted for the exemplative case of ⟨*N*⟩ = 1.6 corresponding to *n*_X_(*t*=0) = 0.32 and *n*_XX_(*t*=0) = 0.26 (vide infra) and used representative decay rates, *k*_R__,X_ = 0.1 ns^–1^ and *k*_R__,XX_ = 4*× k*_R__,X_ = 0.4 ns^–1^. The corresponding τ_EFF_ obtained as the weighted harmonic average of the X and XX lifetimes (see caption of Table 1), integrated emission intensity, and CTR are shown in Figure 4d–f, respectively. The acceleration effect of AR on the XX lifetime is evident in both Figure 4b,c with the noticeable difference that, in the case of nonionizing AR (Figure 4b), quenching of XX by increasingly more efficient AR generates additional X species, which results in AR-independent single exciton emission intensity represented by the long-lived tail. Ionizing AR, on the other hand, leads to a net loss of a photocarrier by a NC which hinders the further formation of a single exciton, resulting in a gradual decrease of the X emission intensity with increasing Φ_AR_. As a result, *τ*_EFF_ decreases more steeply with an increase in Φ_AR_ when AR leads to particle ionization (Figure 4d). Fundamentally, however, despite the dynamic interplay between the two excitonic species, both types of AR lower the total integrated emission intensity to a similar extent, as they accelerate the effective lifetime (Figure 4e), resulting in a CTR that is very weakly dependent on Φ_AR_. We clarify that, in order to provide realistic values for the simulated CTR, we scaled the simulated integrated emission intensities so that the value for Φ_AR_ = 0.9 for nonionizing AR coincided with the estimated *N* = 2323 of our 0.8 wt % nanocomposite (see right axis in Figure 4e and Table 1), which featured comparably efficient AR. This choice was motivated by the qualitative match between the simulation and the TA curve acquired for ⟨*N*⟩ = 1.6 (inset of Figure 4b) suggesting that AR does not lead to the release of electrons in vacuum, which is also consistent with the CB width of CsPbBr_3_ (∼3.5 eV; also indicated as electron affinity “EA” in figure) largely exceeding the bandgap energy of our NCs (∼2.4 eV). Essentially identical trends are found using ⟨*N*⟩ = 2 corresponding to *n*_X_(*t*=0) = *n*_XX_(*t*=0). The near invariance of the timing performance with Φ_AR_ provides important degrees of freedom for optimizing the efficiency of NC-based scintillators by increasing the stopping power and/or optical quality without incurring concomitant losses due to increased AR. For example, increasing the NC density in a nanocomposite scintillator is key to increasing the average *Z* and hence the interaction probability with ionizing radiation. However, this is often accompanied by the formation of large NC agglomerates, which can lead to significant scattering losses of scintillation photons, reducing the selective outcoupling to photodetectors coupled to the scintillator body and potentially increasing self-absorption effects by increasing the average propagation path length within the scintillator. In such a case, it might be advantageous to use more easily dispersible small NCs compared to larger particles, since the intrinsic timing behavior of the scintillator would not be affected by an increased AR rate in small NCs (*k*_AR_ scales universally with the inverse of the volume of a particle),^96,99^ but the technological performance of the radiation detector could be significantly improved due to an enhanced light collection.

**Figure 4:**
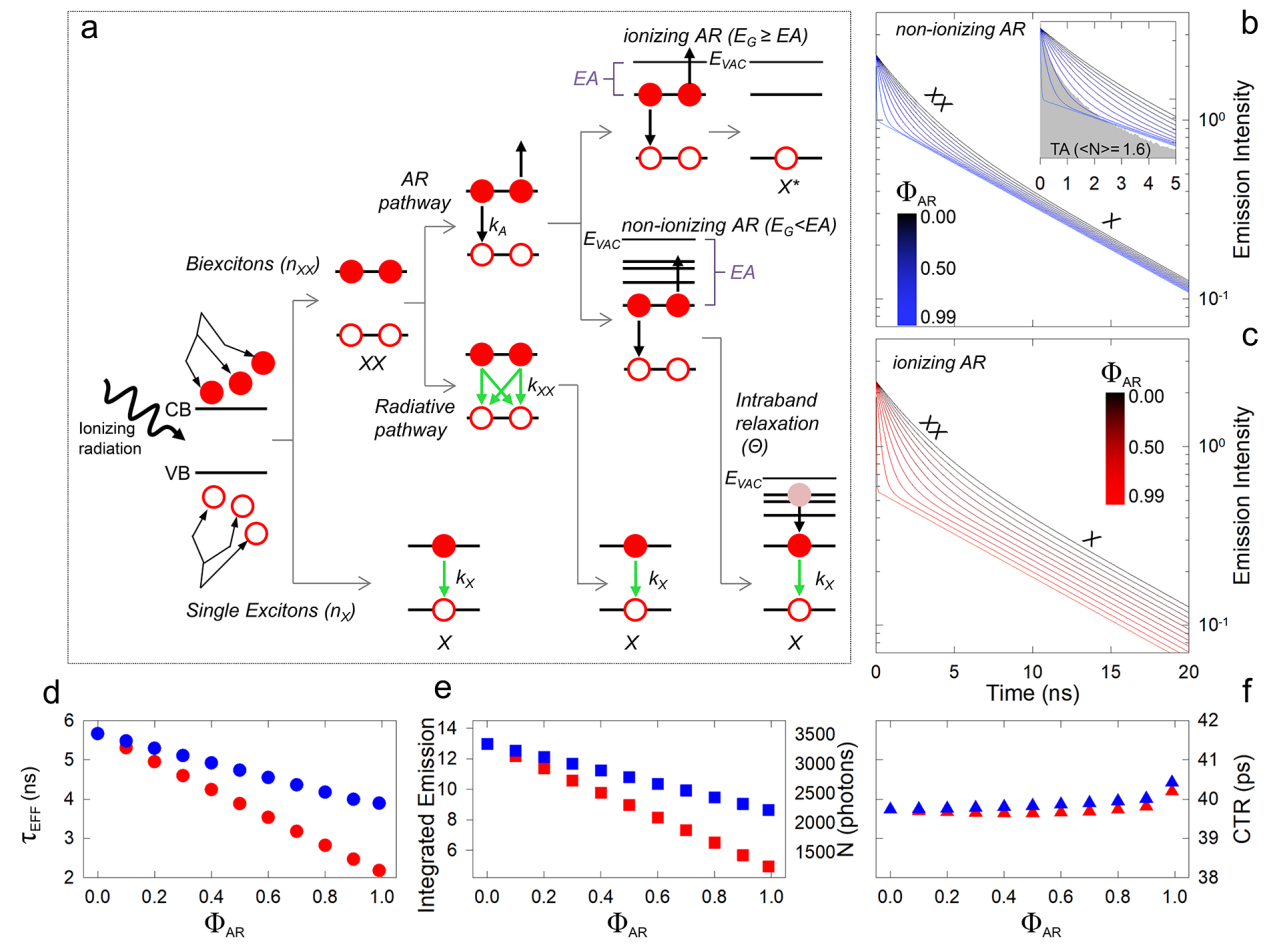
(a) Schematic representation of the possible decay pathways for single (X) and biexciton (XX) states in the presence of AR. Other nonradiative channels are neglected for simplicity. Simulated emission decay curves for ⟨*N*⟩ = 1.6 corresponding to *n*_X_(*t*=0) = 0.32 and *n*_XX_(*t*=0) = 0.26 for (b) nonionizing or (c) ionizing AR. The inset in panel b highlights the similarity between the experimental TA kinetics for ⟨*N*⟩ = 1.6 and the simulation for Φ_AR_ = 0.9 suggesting that in our case AR is mostly nonionizing. (d) Effective emission lifetime, *τ*_EFF_, (e) integrated emission, and (f) CTR extracted from the decay curves in panels a and b. The blue (red) symbols correspond to nonionizing (ionizing) AR.

In this context, it is worth noting that for fixed scintillation kinetics, the CTR of NC-based nanocomposites could be significantly improved by increasing the LY by increasing the NC loading, eventually reaching such high densities that individual particles become sources of secondary excitation of other NCs through their electromagnetic shower released after primary interaction events. However, for most fast-emitting NCs of direct bandgap semiconductors, including CsPbBr_3_ NCs, this requires overcoming the limitation represented by the reabsorption of the scintillation light due to their typically small Stokes shift. In a methodological perspective, it is worth noting that the significant role of AR on the scintillation LY (Figure 4e) suggests particular caution when comparing the scintillation efficiency of NCs of different size or shape and, in the particular case of ionic systems such as LHP NCs, which have a strong tendency to coalesce into large agglomerates in the solid or film state, specific aggregation or processing conditions that could lead to different degrees of quantum confinement and hence different AR efficiencies. Finally, we point out that despite the apparent insignificance of AR for timing performance due to the compensating effects of average lifetime acceleration and LY quenching, other parasitic processes such as ultrafast trapping, which typically occurs in 1–10 ps, are very likely to be overall negative for the LY, since they “statically” quench scintillation on a time scale much faster than the recombination of either exciton type (X or XX), resulting in a net loss of LY without a concomitant acceleration of timing.

In summary, we have developed a low-cost/low-waste approach to fabricate large-scale nanocomposite scintillators with near unity emission efficiency, high radiation hardness, and ultrafast scintillation kinetics due to the recombination of charged-exciton/multiexciton states formed under ionizing excitation. These results elucidate fundamental processes in LHP-NC-based scintillators and provide useful guidelines for future advances in nanocomposite scintillators for radiation detectors and fast timing applications.

## Supporting Information Available

Experimental details; TEM images and size distributions of CsPbBr_3_ nanocrystals; EDS elemental analysis; optical characterization of CsPbBr_3_ nanocomposites *i*) during the polymerization process, *ii*) at increasing concentration of CsPbBr_3_ nanocrystals, and *iii*) at increasing ^60^Co γ-ray dose; structural characterization with powder X-ray diffraction; statistical analysis of TA signal at increasing pump fluences and optical absorption cross section calculation; CTR simulation model; ^1^H nuclear magnetic resonance (NMR) spectra; schematic of NC synthesis with excess solvent recovery and recycle together with TGA, TEM and optical characterization of NCs prepared with recovered wastes; γ-ray irradiation certification and dosimetric certification (PDF)
