# Supplementary material for: Ultrafast and Radiation-Hard Lead Halide Perovskite Nanocomposite Scintillators
Source: ACS Energy Lett. 2023 Aug 28;8(9):3883–94. doi: 10.1021/acsenergylett.3c01396 (PMC10497040; doi:10.1021/acsenergylett.3c01396)
Supplement: Supplementary file 1 — nz3c01396_si_001.pdf [file nz3c01396_si_001.pdf]

Supporting Information

Ultra-fast and radiation-hard lead halide perovskite nanocomposite scintillators

Andrea Erroi[1], Sara Mecca[1], Matteo L. Zaffalon[1], Isabel Frank[2,3], Francesco Carulli[1], Alessia Cemmi[4], Ilaria Di Sarcina[4], Doriana Debellis[5], Francesca Rossi[6], Francesca Cova[1], Kristof Pauwels[7], Michele Mauri[1], Jacopo Perego[1], Valerio Pinchetti[1,8], Angiolina Comotti[1], Francesco Meinardi[1], Anna Vedda[1], Etiennette Auffray[2], Luca Beverina[1] and Sergio Brovelli[1]*

[1] *Dipartimento di Scienza dei Materiali, Università degli Studi Milano - Bicocca, via R. Cozzi 55,20125 Milan, Italy.*
[2] *CERN, Esplanade des Particules 1, 1211 Meyrin, Switzerland*
[3] *LMU Munich, Geschwister-Scholl-Platz 1, 80539 Munich, Germany*
[4] *ENEA Fusion and technology for nuclear safety and security department, Casaccia R.C., Via Anguillarese 301, 00123 Rome, Italy*
[5] *Electron Microscopy Facility, Istituto Italiano di Tecnologia, 16163 Genova, Italy*
[6] *IMEM-CNR Institute, Parco Area delle Scienze 37/A, 43124 Parma, Italy*
[7] *ESRF - The European Synchrotron, 71 Avenue des Martyrs, 38000 Grenoble, France*
[8] *Current address: Nanotechnology and Advanced Spectroscopy Team, C-PCS, Chemistry Division, Los Alamos National Laboratory, Los Alamos, NM, 87545 USA*
Corresponding author: sergio.brovelli@unimib.it

Methods

Chemicals: Cesium carbonate (Cs_2CO_3, 99%), Lead (II) bromide ($PbBr_2$, 99.99%) were purchased from Fluorochem. Propionic acid (PA, >99.5%), Tetrabutylammonium bromide (TBAB, >98%), Methyl methacrylate (MMA, 98%), Lauryl methacrylate (LMA, 98%), 2,2-Dimethoxy-2-phenylacetophenone (IRGACURE651, 99%) were purchased from Sigma-Aldrich. Oleylamine (OLAM, 90%) was purchased from Acros Organic. Isopropanol (HiPerSolv chromanorm for HPLC, >98%), Heptane (Gpr rectapur, 99.8%) were purchased from VWR. Turbo emulsifier homogenizer was bought from IKA and it is composed of motor group T25/T50 digital ultra turrax and dispersing tool with code S25N–25G and S50N-G45M for volumes respectively until 2 L and above.

Synthesis of native $CsPbBr_3$ NCs. To synthesize 8 g of $CsPbBr_3$ NCs, $PbBr_2$ (60 mmol, 22.02 g) and TBAB (60 mmol, 19.32 g) were dissolved at 80 °C in a mixture of OLAM (540 mmol, 177.6 mL), PA (540 mmol, 40.2 mL) and isopropanol (60 mL). After complete dissolution, the mixture was cooled down to room temperature. A solution of Cs_2CO_3 (6 mmol, 1.95 g) in propionic acid (79.8 mmol, 6 mL) is then prepared and diluted in 3600 mL of a heptane/isopropanol solution (2:1 in volume). As the latter is put under turbo-emulsifier homogenization at 15k RPM, the first solution is swiftly added, inducing a rapid change from colourless to bright yellow. The mixture is let homogenise for 30 s, then mixing is stopped. The final crude solution is clear and brightly luminescent, highlighting the formation of well dispersed nanostructures. Isopropanol (1.8 L) is added to the crude solution and centrifuged at 4500 rpm for 2 min to precipitate the nanostructures. The supernatant was discarded. The recycling protocols of both the volatile solvents and the non-volatile residue are detailed in the Supplementary Information.

Synthesis of $CsPbBr_3$ nanocomposite. To complete the evolution of the NCs and realize the nanocomposite scintillator, the appropriate concentration of native NCs is dispersed in LMA (the amount of LMA depends on the size of the nanocomposite, for devices of 50x50x0.3 cm it is 150

mL) and stirred to wet the NC surface and ensure fine dispersion of the individual NCs. To the colloidal solution is added the secondary monomer, MMA (MMA/LMA 80:20 wt %) and the radical photoinitiator IRGACURE651 (0.33 wt %). After stirring the mixture for 30 min and sonication for 5 min to facilitate NC dispersion, the homogeneous mixture was poured into a mould made of two low-roughness pieces of tempered glass linked by a PVC gasket and irradiated with 365 nm light from an ultraviolet lamp for 2 h to trigger radical polymerization. The polymerization was then completed by keeping samples in dark for 2 h while leaving them in the mould to avoid the creation of cracks. After completion of the procedure, the slabs were removed from the mould.

Structural characterization.

XRD measurements. Powder x-ray diffraction experiments were performed on a Rigaku SmartLab SE diffractometer equipped with Cu Kα radiation, with a Cu kβ radiation filter and a Hy-Pix 400 detector, operating at 40 KV and 30 mA. Data collections were performed in Bragg-Brentano geometry with a scan range between 10° and 50° 2 theta with a step size of 0.02° and scan speed of 0.1° min^{-1}. Powdery samples were gently dispersed on a Silicon crystal sample holder (zero background sample holder).

Transmission electron microscopy. The High-Angle Annular Dark Field (HAADF) images in scanning TEM (STEM) mode and the high-resolution TEM images (HRTEM) of powdered NCs were acquired in a JEOL JEM-2200FS transmission electron microscope equipped with in-column Omega filter, operating at 200 kV. For TEM imaging in bright-field (BF) mode, anular dark-field imaging in scanning transmission electron (ADF-STEM) mode and for energy-dispersive X-ray spectroscopy (EDS) on polymer matrix nanocomposites, the samples were sectioned with an ultramicrotome (Ultracut EM UC6, Leica) equipped with a diamond knife (Diatome). Sections of 70 nm in thickness were collected on a Cu TEM grid, 200 hexagonal mesh; the grid was coated with a thin layer of carbon to preserve the nanocomposite sections and avoid charging problems. BF-TEM imaging was carried out with a JEOL JEM 1011 transmission electron microscope operated at 100 kV. ADF-STEM and EDS analyses were performed using a JEM-1400Plus, with thermionic source (LaB_6), operated at 120kV. The EDS data have been acquired using a JEOL Dry SD30GV silicon-drift detector (SDD), with 30 mm^2 effective area and using an analytical double tilt sample holder. In the EDS analyses the Cu signal is attributable to the TEM grid.

Time Domain NMR. Proton Time Domain NMR (^{1}H TD-NMR) is a technique based on the direct analysis of the NMR Free Induction Decay without Fourier transform. Applications to polymer analysis consider that the functional shape of the FID is related to the local mobility. Polymer chains with more restricted mobility display faster relaxation. Experiments were performed on a Minispec mq20 (Bruker) operating at a Larmor frequency near 20 MHz and equipped with a nitrogen gas fed BVT3000 temperature control unit. Samples were prepared by cutting a piece that could fit a 10 mm external diameter NMR tube without further preparation. To avoid significant loss of signal associated to fast relaxing domains during the instrumental dead time, we applied a MSE refocusing block before acquiring the FID. Increasing temperature enhances the mobility and allows more precise differentiation of similar systems. Thus, we worked at 333 K (60 °C), the highest temperature where the NPs are expected to display long term stability.

The acquired FID was analysed with the following equation:

$$\frac{FID(t)}{FID(0)} = f_r e^{-\left({}^{t}/_{T_{2r}}\right)^2} + (1 - f_r) e^{-\left({}^{t}/_{T_{2m}}\right)}$$

Where f_r is the rigid fraction, T_{2r} and T_{2m} are the relaxation times associated with the rigid and mobile phases respectively.

γ-ray irradiation experiments. Similar portions of the same nanocomposite sample were placed in individual polypropylene sealed vials whose gamma ray attenuation is negligible (Eppendorf Tubes), one for each dose. The vials were irradiated in a pool-type gamma irradiation chamber equipped with a ^{60}Co (mean energy ~1.25 MeV) γ-source rods array, uniformly irradiating the composite at 3.05 kGy_{air}/h dose rate value. Nanocomposite samples were irradiated at different cumulated absorbed doses up to 1 MGy by varying the irradiation time. The irradiation has been carried out at the CALLIOPE Gamma Irradiation Facility at ENEA Casaccia Research Centre (Rome). Throughout the paper, the given dose is in air.

Optical Measurements. Optical absorption measurements were measured in hexane with a Cary 50 UV–Vis spectrophotometer. Photoluminescence quantum yield measurements were performed using and an integrating sphere coupled to a spectrometer and a charge-coupled device. The PL spectra were excited using a laser source at 3.06 eV (405 nm); the emitted light was collected using a custom apparatus featuring a liquid nitrogen-cooled, back-illuminated, and UV-enhanced charge-coupled device detector (Jobin-Yvon Symphony II) coupled to a monochromator (Jobin-Yvon Triax 180) with 100 lines/mm gratings and corrected for the spectral response of the system. Time-resolved PL were carried out using 3.06 eV (405 nm) ps-pulsed diode lasers (Edinburgh EPL405, ∼70 ps pulses); the emitted light was collected with a phototube coupled to a Cornerston 260 1/4 m VIS-NIR Monochromator (ORIEL) and a time-correlated single-photon counting unit (time resolution ∼400 ps).

Ultrafast transient absorption spectroscopy measurements were performed on Ultrafast Systems' Helios TA spectrometer. The laser source was a 10 W Hyperion amplified laser operated at 2.142 kHz producing ~260 fs pulses at 1030 nm and coupled with an independently tunable APOLLO-Y optical parametric amplifier from the same supplier that produced the excitation pulses at 2.69 eV. After passing the pump beam through a synchronous chopper phase-locked to the pulse train (1.071 kHz, blocking every other pump pulse), the pump fluence on the sample was modulated from 1.5 μJ cm^{-2} to 600 μJ cm^{-2}. The probe beam was a white light supercontinuum.

Radioluminescence measurements. $CsPbBr_3$-based composites were excited by unfiltered X-ray irradiation using a Philips PW2274 X-ray tube, with a tungsten target, equipped with a beryllium window and operated at 20 kV. At this operating voltage, a continuous X-ray spectrum is produced by a Bremsstrahlung mechanism superimposed to the L and M transition lines of tungsten, due to the impact of electrons generated through thermionic effect and accelerated onto a tungsten target. The RL was collected using the same custom apparatus used for the PL measurements.

Light Yield measurements. The LY values were evaluated using relative and absolute methods. The relative LY is obtained by direct comparison of the RL response of nanocomposites and commercial EJ276D plastic scintillator of the same size used as reference, with a LY of 8600 photons/MeV. The RL is collected using the setup described above. The absolute LY measurements were performed exposing the samples to an X-ray generator (model XRG3D from Inel) operated at 25 kV and equipped with a molybdenum anode and a zirconium filter (100 µm thickness). Under the experimental conditions, the integrated flux is 1.10×10^6 photons/mm²/s and the average energy is 15.4 keV, more details are provided in ref.[97]. The samples were coupled to a sCMOS sensor with a fiber optic plate (FOP) coupling (model Zyla5.5 HF from Andor). The images were acquired in binning 4x4 and an exposure time of 30 s and converted into scintillating photons per incoming X-ray based on a pixel size of 6.5 µm, an electronic gain of 0.46 e/ADU and a quantum efficiency of 17 %. The

light collection was done from the face opposite to the X-ray beam. The X-ray absorption efficiency of the samples was estimated by imaging the samples with a scintillator fully absorbing the X-ray beam (500 µm thick CsI). The ratio between the acquired images without samples (flat-field images) and the ones acquired with samples partly blocking the X-ray beam estimates the fraction of transmitted X-rays. The average energy deposited in the sample is then obtained by combining the average energy of the X-rays and the X-ray absorption efficiency of the samples. The previously described measurement estimates the scintillation light emitted in the forward cone, the refraction index of the sample then permits estimating the light emitted isotropically. A reference sample BGO (500 µm) was also measured to help the comparison.

Time-resolved luminescence spectroscopy with synchrotron excitation: Time-resolved photoluminescence measurements were carried out in ultra-high vacuum with synchrotron radiation at the Superlumi experimental station at DESY (Hamburg, Germany). The selected excitation energies spanned from 3.7 eV to 40 eV using 0.3 nm bandwidth, 130 ps pulse width, and 16 ns interpulse. The emitted light was collected with a Hamamatsu H8259-01 phototube coupled to time-correlated single-photon counting unit (time resolution ~400 ps) and an Andor Kymera 328i monochromator equipped with 300 lines/mm and 500 nm blaze grating.

Time-resolved scintillation experiments. Samples were excited with a Hamamatsu XRT N5084 pulsed tungsten X-ray tube operating at 40 keV, where a PicoQuant PDL 800-B pulsed diode laser with 40 ps pulse width (full-width-at-half-maximum - FWHM) acts as excitation source of the X-ray tube. The energy spectrum of the produced X-rays ranges from 0 to 40 keV with a pronounced peak between 9 and 10 keV, due to tungsten L-characteristic X-ray, and mean energy of about 15 keV. The X-rays hit the sample after crossing a brass collimator. The scintillation light is collected in TCSPC by a Becker & Hickl HPM 100-07 hybrid photomultiplier tube (HPM). The signal of the HPM was processed by an ORTEC 9237 amplifier and timing discriminator and acted as stop signal at a Cronologic xTDC4 time-to-digital-converter (TDC). The start signal was given by the external trigger of the pulsed laser. The overall impulse response function (IRF) of the system was obtained as the analytical convolution between the measured IRF of the laser together with HPM and the IRF of the X-ray tube, resulting in around 160 ps FWHM. The RL was spectrally selected using an optical bandpass filter at 500 nm with 40 nm FWHM mounted to the HPM that removed parasitic contributions due to air excitation by X-rays.

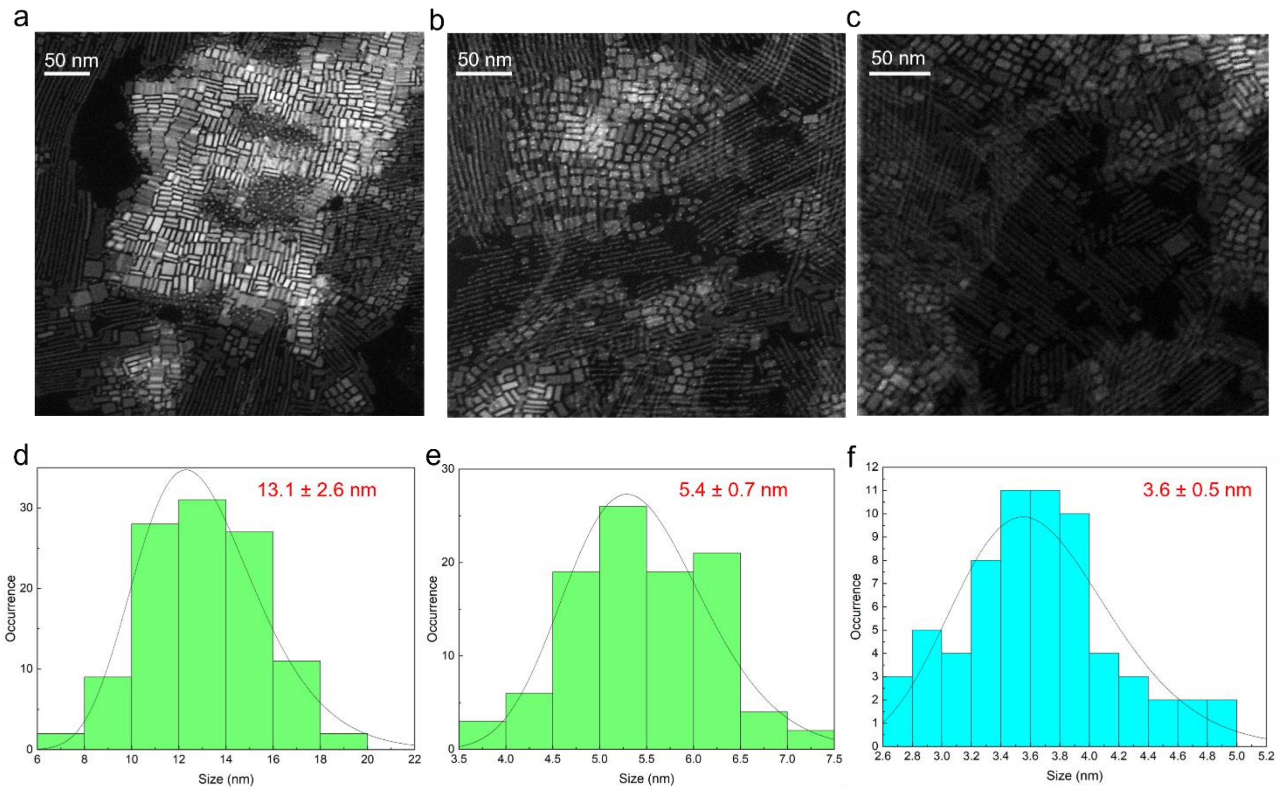

Figure S1. STEM images of native $CsPbBr_3$ nanostructures **(a,b)** and a zoom in STEM image on nanowires-like structures **(c)**. Size distributions of nanoplatelets-like $CsPbBr_3$ (side: 13.1±2.6 nm, thickness: 5.4±0.7 nm) – green histogram – **(d,e)** and nanowires-like $CsPbBr_3$ (thickness: 3.6±0.5 nm) – cyan histogram – **(f)** as extracted from **(a,b)** and **(c)**, respectively.

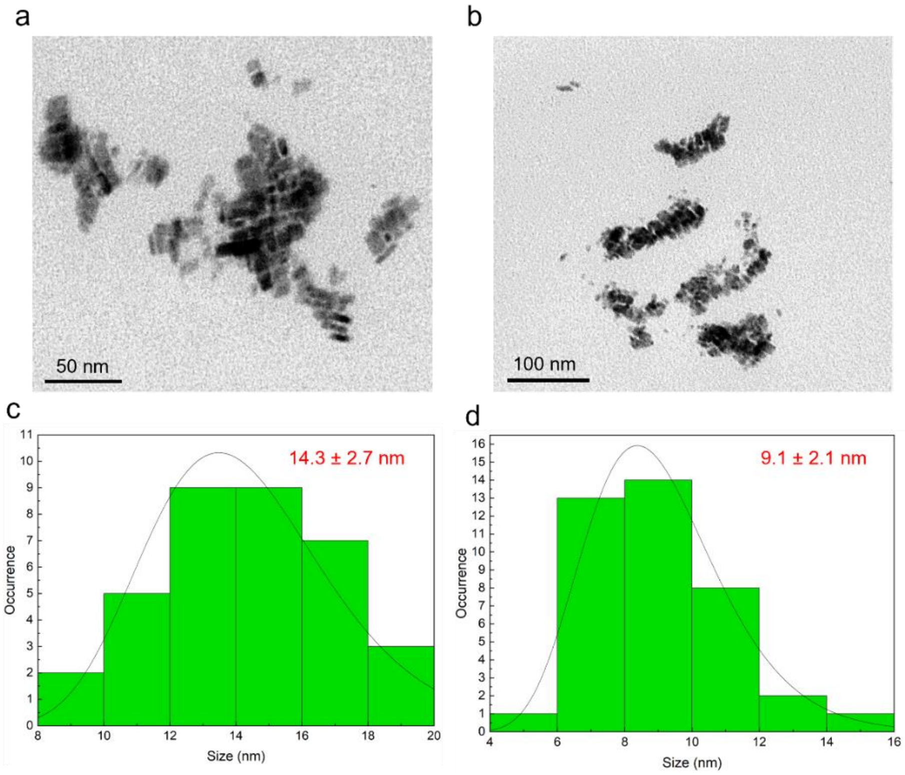

Figure S2. BF-TEM images of $CsPbBr_3$ polymer nanocomposite **(a,b)**. Size distributions of $CsPbBr_3$ (side: 14.3±2.7 nm, thickness: 9.1±2.1 nm) – green histogram – **(c,d)** as extracted from **(a,b)**.

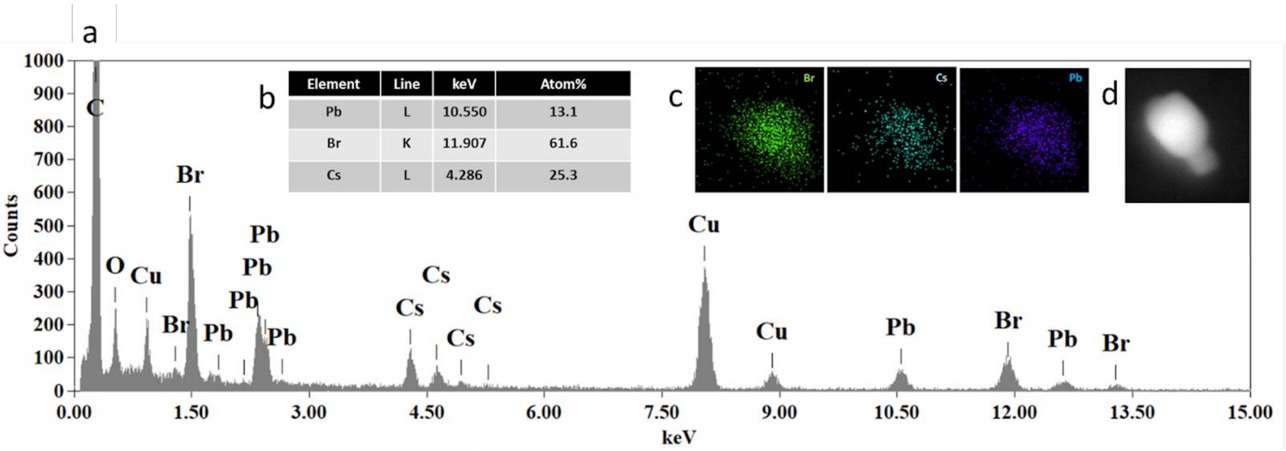

Figure S3. ADF-STEM/EDS data of the $CsPbBr_3$ Cs in polyacrylate matrix. EDS spectra **(a)**, chemical composition **(b)** and elemental distribution **(c)** of the analysed area **(d)**.

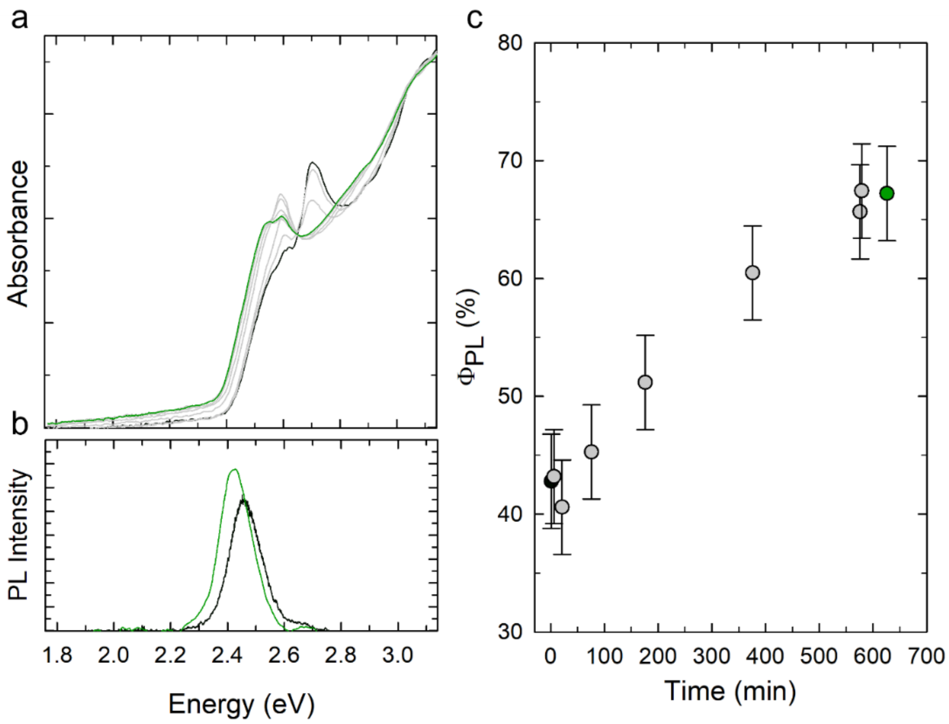

Figure S4. a, Optical absorption spectra collected during the NC evolution in a mixture of acrylate monomers, same as used for the polymeric nanocomposite but without UV illumination and polymerization initiators. The corresponding PL spectra are shown in '**b**' at the begin (t = 0 min, black line) and at the end (t = 650 min, green line) of the NC evolution process. **c,** PL quantum yield values of the same NCs solution measured during the evolution process.

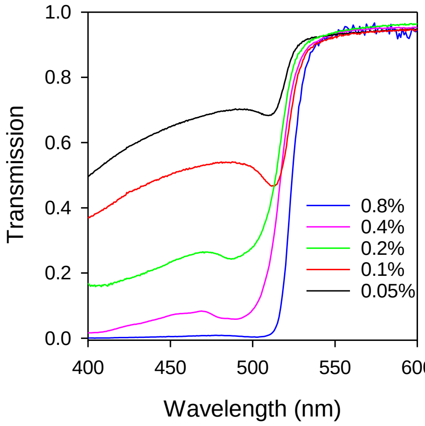

Figure S5. Optical transmission spectra of polyacrylate nanocomposites embedding increasing concentration of $CsPbBr_3$ NCs.

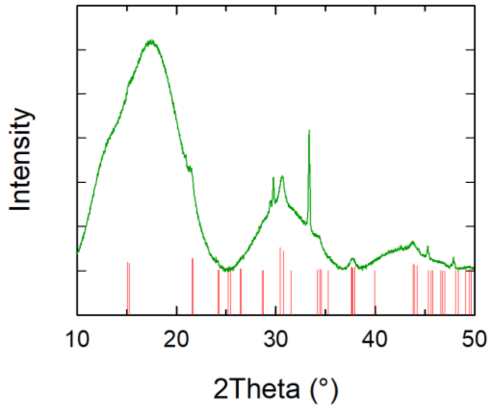

Figure S6. Powder X-ray diffraction pattern of the NCs embedded in the nanocomposite (green line) without subtraction of the signal due to the polymer matrix and the calculated PXRD pattern for orthorhombic $CsPbBr_3$ (red ticks, ICSD 97851).

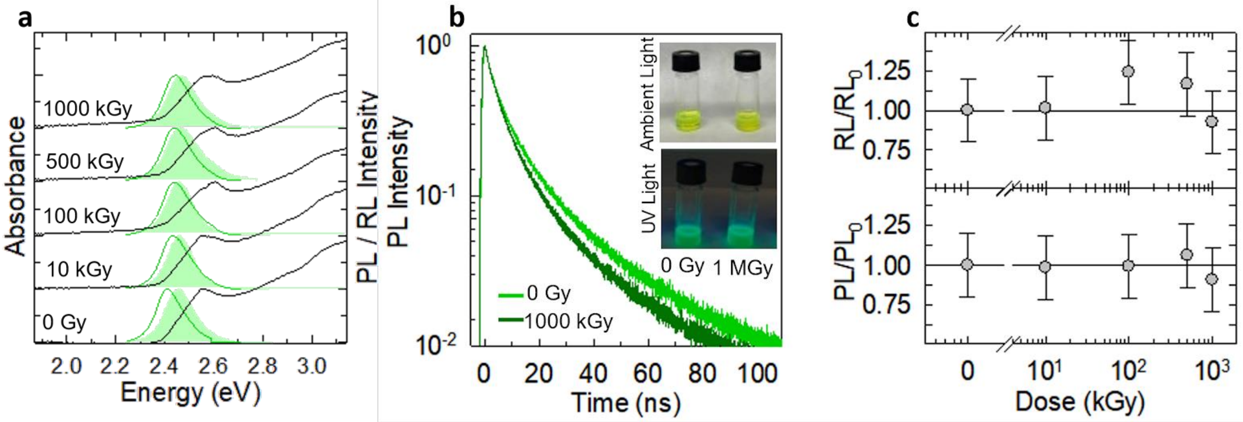

Figure S7 a, Optical absorption (black line), PL (green line), and RL (shaded area) spectra of $CsPbBr_3$ nanocrystals dispersed in acrylate monomer solution at increasing γ-ray doses as indicated in figure. All the spectra have been normalized and vertically shifted for clarity. **b,** Normalized PL decay traces of $CsPbBr_3$ NCs in monomer solution at 0 Gy and 1000 kGy doses. Inset: photographs of the NCs solutions under ambient and UV light for the non-irradiated and the most irradiated samples. **c,** RL intensity (top panel) and PL intensity (bottom panel) of the same $CsPbBr_3$ NCs solutions normalized at their intensity before γ-ray irradiation (RL_0 and PL_0).

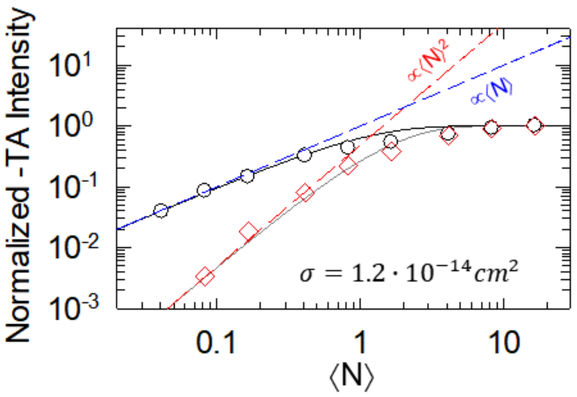

Figure S8 Pump-fluence dependence of the single exciton TA amplitude (black circles) evaluated from the late-time TA amplitude (~4 ns), when there are virtually no contributions from short-lived multiexcitons and each photoexcited NC is populated with a single exciton independent of its initial occupancy. This is further corroborated by the linear growth rate with ⟨N⟩ (dashed blue line) before single exciton state filling occurs. The amplitude of the multi-excitonic component (red diamonds) is obtained by subtracting the TA amplitude relative to single excitons from the total TA intensities at early times following the method introduced in ref.[1]. At low pump fluences (⟨N⟩ < 0.5), the multi-excitonic component follows a quadratic scaling with $\langle N\rangle^2$, as expected for biexcitons (dashed red line). The solid lines represent their respective fits assuming a Poisson statistic of the NC occupancy with the same absorption cross-section value for both fits ($\sigma = 1.2\cdot10^{-14}$ cm^2). The obtained value for σ is also in good agreement with previous reports on CsPbBr3 NCs the of similar size (14x14x9 nm^3, as extracted from Figure S2), consistent with the linear scaling of σ with NC volume.

- **CTR Simulation:** In order to evaluate the expected CTR as a function of the Auger Recombination efficiency (Φ_{AR}) we developed a simplified model which describes all the possible recombination mechanisms involved in $CsPbBr_3$ NCs containing single excitons and biexcitons. Considering the decay pathways for single (X) and biexciton (XX) states in the presence of AR, as shown in **Figure 4a** of the manuscript, we evaluate the time evolution of the population solving the following rate equations:

$$\dot{n_{XX}} = -n_{XX}\left(k_{R,XX} + k_{AR}\right) \quad \text{(Eq.1)}$$

$$\dot{n_X} = -n_X k_{R,X} + n_{XX}\left(k_{AR}\Theta + k_{R,XX}\right) \quad \text{(Eq.2)}$$

The solutions of the equation rate for biexcitons and single excitons are given respectively by:

$$n_{XX}(t) = n_{XX}^0 e^{-(k_{R,XX}+k_{AR})t} \quad \text{(Eq.3)}$$

$$n_X(t) = \left[n_X^0 + n_{XX}^0 \frac{\Theta k_{AR}+k_{R,XX}}{k_{R,XX}+k_{AR}-k_{R,X}}\left(1 - e^{-(k_{R,XX}+k_{AR}-k_{R,X})t}\right)\right] e^{-(k_{R,X})t} \quad \text{(Eq.4)}$$

Where $n^0{}_X$ (0.323) and $n^0{}_{XX}$ (0.258) represent the initial excitons and biexcitons population, calculated from the Poisson distribution with the average occupancy value of ⟨N⟩=1.6 (which was determined by comparing the sample scintillation kinetics measurements under X-ray stimulation with the TA dynamics collected at different exciton population). The other parameters were obtained from experimental measurements on our samples, specifically $k_{R,X} = 10^8$ s^{-1}, $k_{R,XX} = 4 \cdot k_{R,X} = 4 \cdot 10^8$ s^{-1} and QY=0.9. The Θ parameter was introduced to discriminate the case of ionizing or non-ionizing Auger Recombination, where (1- Θ) is the probability of particle ionization by carrier ejection or by trapping of hot carriers.

The first term in the brackets of **Eq. 4** describe the time evolution of the "native" single exciton population, while the second represents the excitons population generated by biexcitons radiative recombination and Auger recombination (when Θ≠1). The kinetics of biexcitons and excitons populations, in case of ionizing and non-ionizing Auger Recombination, as a function of Φ_{AR} are reported in plot in Figure S9a, b and c, respectively.

From these equations we simulated the emission kinetics, reported in plot in Figure 4d,e from which we could extract the effective scintillation lifetime, τ_{EFF} (calculated as the weighted harmonic average of the time constants of the two decay contributions) and the average number of photons emitted per single NC (calculated as the integrated profile of the emission decay) which is proportional to the light output, N.

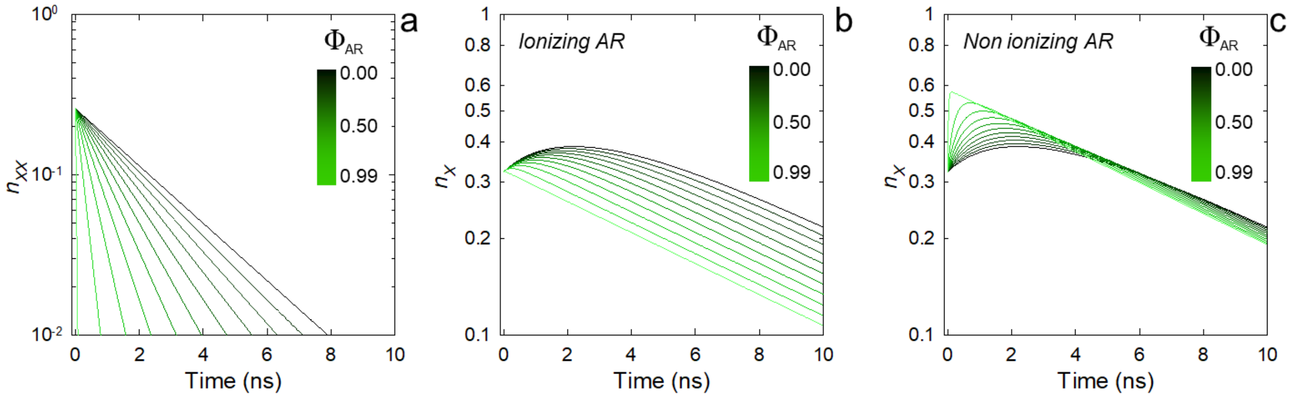

Figure S9 Time evolution of the population of biexcitons **(a)** and single excitons in case of ionizing **(b)** and non-ionizing **(c)** Auger Recombination as a function of the AR efficiency.

- **Recovery and recycle of excess reagents and solvents.**

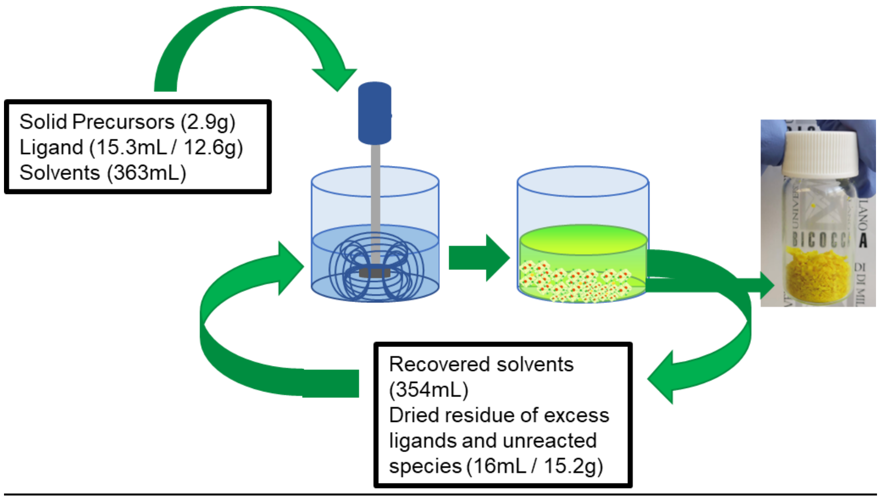

Figure S10 Schematic representation of the "circular" synthetic process, that takes advantage of wastes reuse, with specific quantities involved in synthesis of a 240 mL scale batch.

<table>
<tr><th>Species</th><th>Employed amount</th><th>Total employed amount</th><th>Recovered amount</th></tr>
<tr><td>Solid reactants</td><td>2.9 g</td><td rowspan="2">15.6 g</td><td rowspan="2">15.7 g</td></tr>
<tr><td>Ligands</td><td>12.6 g</td></tr>
<tr><td>Solvents</td><td>363 mL</td><td>363 mL</td><td>354 mL</td></tr>
</table>

Table 1 Total amount of employed and recovered different species on batch scale of 240 mL.

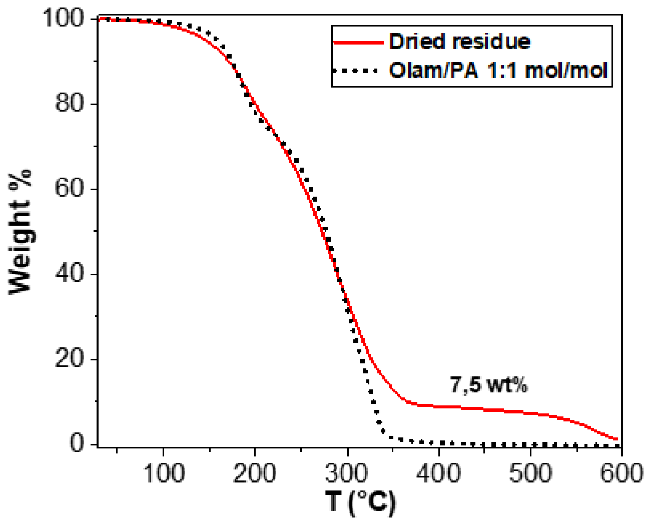

Figure S11 Thermogravimetrical analysis (TGA) of the dried residue with respect to ligands mixture.

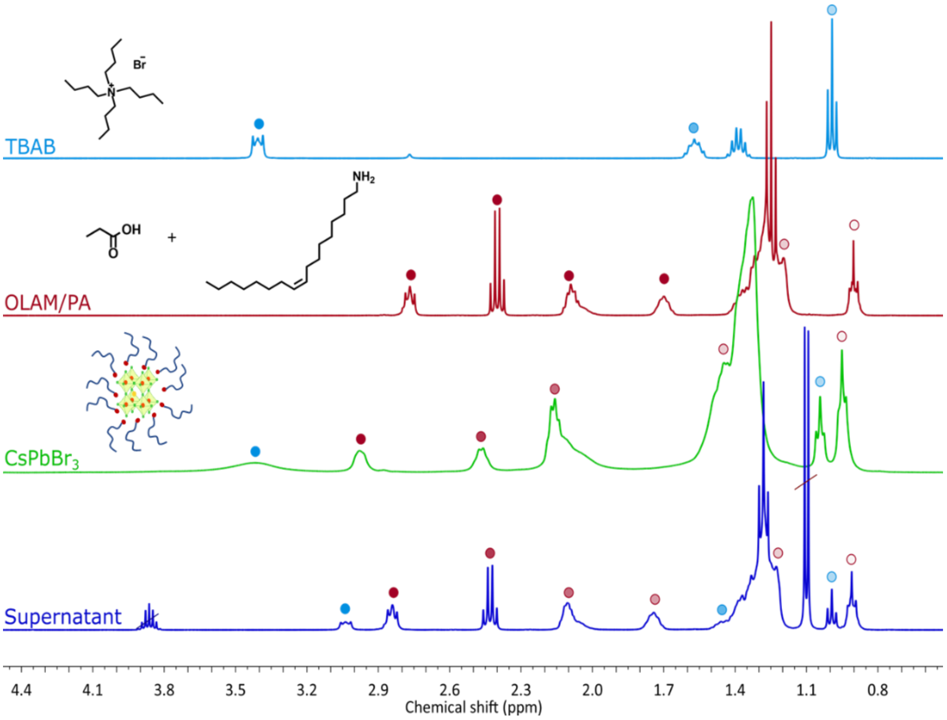

Figure S12 ^{1}H nuclear magnetic resonance (NMR) in C_6D_6 of respectively: tetrabutylammonium bromide (TBAB), oleylamine (OAm) / propionic acid (PA) 1:1 mol ratio mixture, $CsPbBr_3$ sample and dried supernatant from synthesis.

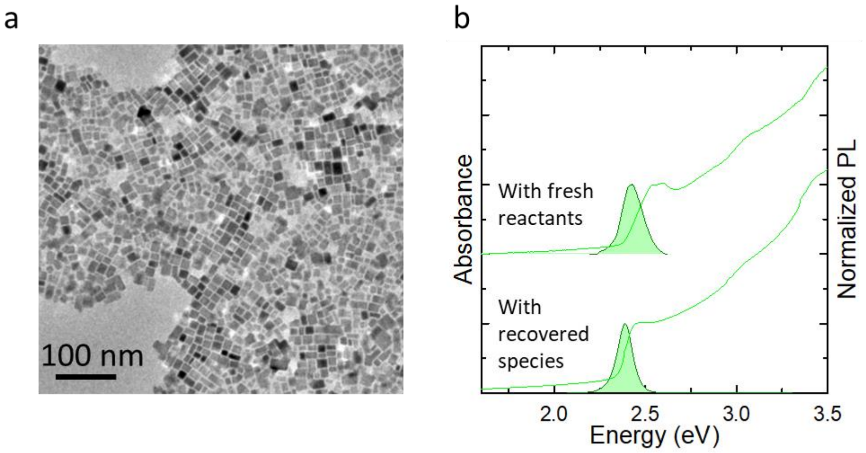

Figure S13 (**a**)**,** HRTEM image of $CsPbBr_3$ NCs prepared with recovered wastes and (**b**)**,** normalized absorption (curve) and photoluminescence (shaded area) spectra of the same in comparison with NCs obtained with fresh reactants.

Considering a reaction scale performed on a 240 mL batch (total amount of solvents used 263 mL), we collected 245 mL of a first fraction distilling at 75 °C and 20 mL of a second fraction distilling at 83 °C. Since heptane (Hept) and isopropanol (iPrOH), the only two solvents used, have an azeotrope concentration of 46:56 v/v with a boiling point of 76.4°C (1 atm), we recovered 265 ml of Hept/iPrOH 1:1.3 v/v. Gas chromatography – mass spectrometry (GC-MS) analysis showed no impurities in the recovered solvent mix, allowing its reuse for further synthesis. The dried, non-volatile, viscous residue (16 g) is initially homogeneous and then a white solid precipitate overnight. This precipitate can be re-dispersed by heating the oily residue under magnetic stirring at 80 °C: this solid is therefore probably composed of $[PbBr_6]^{4-}$ aggregates and $CsPbBr_3$ clusters which, due to their very small dimensions, were not recovered during the previous centrifugation step. This dried residue composition was characterised by thermogravimetric analysis - TGA (7 wt% of the residue at 490 °C, Figure S11), ^{1}H nuclear magnetic resonance - NMR- in solution (C_6D_6, to evaluate the relative amount of OAm, PA and TBAB, comparable to the feed ratio, Figure S12) and inductively coupled plasma optical emission spectroscopy - ICP-OES (Pb 2.25 ± 0.05 wt% and Cs 0.04 ± 0.02 wt%). We repeated the aforementioned synthetic procedure on a 120 mL scale with recovered solvents and non-volatile residue, adding only the required amount of fresh reactants. Specifically, 68.8 mL of distilled recovered solvents (iPrOH/Hept 1.3:1 v/v), to which we added 51 mL of fresh heptane to obtain the desired Hept/iPrOH 2:1 ratio, were used to dilute Cs_2CO_3 (62 mg, 0.19 mmol, instead of 65 mg) previously dissolved in 0.1 mL of propionic acid (1.3 mmol). We used 6.6 mL of the non-volatile recovered mixture (6.27 g) and added $PbBr_2$ (438 mg, 1.32 mmol), TBAB (205 mg, 0.6 mmol), OAm (1.1 mL, 3.3 mmol) and iPrOH (1 mL). The resulting dispersion was dissolved under stirring at 80 °C and then cooled to room temperature. The two solutions were then mixed under turbo-emulsification and further homogenised for 30 s. The dispersion thus obtained was diluted with iPrOH (60 mL) and centrifuged (4500 rpm, 10 min), obtaining 282 mg of NCs with morphology and steady-state optical properties comparable to those obtained from fresh reagents and solvents after evolution (Figure S11). In principle, the supernatant of this second reaction could be reused.

- **Supporting Documents:**

Irradiation certification of the ENEA Calliope irradiation facility on plastic nanocomposites and nanocrystals (pages 1,2) together with the *dosimetric certification* of the facility (pages 3,4).

ENEA<br>Italian National Agency for New Technologies, Energy and Sustainable Economic Development	Date: April 1st, 2022<br>Prot. ENEA/2022/24690/FSN FISS SNI	Ref. Doc.<br>Prot. ENEA/2021/11982/FSN FISS	Irradiation Certification number:<br>1/2022<br>Pag. 1/4
Calliope Irradiation Plant<br>ENEA Casaccia R.C.	Irradiation Plant Technician	Calliope Irradiation Plant Director	

IRRADIATION CERTIFICATION

Sample description: Nanoscintillators.

Customer: Milano Bicocca University.

Reference documents: N°19 - 12/11/2020, N°13 - 27/10/2020.

DOSIMETRIC CERTIFICATION REFERENCES: N°19 - 12/11/2020, N°13 - 27/10/2020

IRRADIATION TIME:

**Irradiation start:**	18:00 02/03/2021
**Irradiation stop:**	14:00 16/03/2021

ENVIRONMENTAL CONDITIONS IN THE IRRADIATION CELL:

**Temperature:**	25 °C
**Pressure:**	1 atm
**Atmosphere:**	air

Note : **The dose rate used is the average of all dosimetric values <$\dot{D}_{water}$> reported in the dosimetric certificate. The dose rate used in the irradiation test takes into account the natural decay of ^{60}Co radioisotopic source. The management of the irradiation test is carried out by using a Calliope plant dedicated software that allows a daily dose rate value update, the irradiation time determination and the absorbed dose calculation.**

STANDARD REFERENCE REQUESTED:

IRRADIATION CERTIFICATION

Nanoscintillators

Step	Dose rate at the beginning of irradiation [Gy_{air}/h]	Dose rate at the end of irradiation [Gy_{air}/h]	Irradiation time (hh:mm:ss)	Absorbed dose [Gy_{air}]
1	843.78	843.48	15:19:54	12933.66
2	3013.44	2999.37	289:44:38	871044.35
3	3003.69	2999.37	93:14:15	279850.49
TOTAL			398:18:47	1163828.50

Nota:

- the dose rate value was obtained by updating, at the beginning of the irradiation test, the value reported in the dosimetric certification (N°19 - 12/11/2020, N°13 - 27/10/2020);
- the absorbed dose relative error is 2.5% for step 1, and 5% for step 2-3, and the absorbed dose value is referred to air.

DOSIMETRIC CERTIFICATION

Date of dosimetric measurements: N°19 - 12/11/2020.

Equipment: Spectrofotometer UV/Vis Perkin Elmer Lambda 950

Dosimeter :

Alanine	
Fricke	X
Red Perspex	
TLD	

Dosimetric measurements have been carried out according to ENEA TDI 87011A document.
The relative error associated to the different dosimeters is:

Alanine:	4%
Fricke	2.5%
Red Perspex:	5%
TLD:	10%

DOSIMETRIC RESULTS

Dosimeter number	Irradiation time (hh:mm:ss)	Absorbed dose/D_{water} [Gy]	Dose rate/$\dot{D}_{water}$ [Gy/h]
1	00:10:00	161.23	967.43
2	00:10:00	160.28	961.70
3	00:10:00	167.69	1006.15
4	00:10:00	161.62	969.74
5	00:10:00	162.93	977.60

Average of all dosimetric values

$<\dot{D}_{water}> = 938.58$ Gy/h

$<\dot{D}_{air}> = 0.899 \times <\dot{D}_{water}> = 843.78\ Gy_{air}/h$

Italian National Agency for New Technologies, Energy and Sustainable Economic Development	Date: March 31st, 2022<br>Prot. ENEA/2022/ /FSN FISS SNI	Ref. Doc.<br>Prot. ENEA/2021/11982/FSN FISS	Irradiation Certification number:<br>1/2022<br>Pag. 34/4
Calliope Irradiation Plant<br>ENEA Casaccia R.C.	Irradiation Plant Technician	Calliope Irradiation Plant Director	

DOSIMETRIC CERTIFICATION

Date of dosimetric measurements: N°13 - 27/10/2020.

Equipment: Spectrofotometer UV/Vis Perkin Elmer Lambda 950

Dosimeter :

Alanine

Fricke

Red Perspex X

TLD

Dosimetric measurements have been carried out according to ENEA TDI 87011A document.
The relative error associated to the different dosimeters is:

Alanine: 4%
Fricke 2.5%
Red Perspex: 5%
TLD: 10%

DOSIMETRIC RESULTS

Dosimeter number	Irradiation time (hh:mm:ss)	Absorbed dose/D_{water} [Gy]	Dose rate/$\dot{D}_{water}$ [Gy/h]
1	03:00:00	10625.00	3541.67
2	03:00:00	10648.00	3476.00

Average of all dosimetric values

Average of all dosimetric values

$<\dot{D}_{water}> = 3351.99$ Gy/h

$<\dot{D}_{air}> = 0.899 \times <\dot{D}_{water}> = 3013.44\ Gy_{air}/h$

Supporting References

1. Makarov NS, Guo S, Isaienko O, Liu W, Robel I, Klimov VI. Spectral and Dynamical Properties of Single Excitons, Biexcitons, and Trions in Cesium-Lead-Halide Perovskite Quantum Dots. *Nano letters* 2016, **16**(4)**:** 2349-2362.
